# Antibiotics administration during weaning ameliorates intestinal mucosal inflammation in adult mice and their offspring

**DOI:** 10.3389/fimmu.2026.1741596

**Published:** 2026-02-26

**Authors:** Junyang Cao, Zhanjun Lu, Xiulong Xia, Fengwanni Wang, Changqin Liu, Wenqing Zhou, Zhanju Liu, Jiali Deng, Xiaoming Hu

**Affiliations:** 1 School of Life Science, Shanghai University, Shanghai, China; 2 Health Science Center, East China Normal University, Shanghai, China; 3 Department of Gastroenterology, Shanghai General Hospital, Shanghai Jiao Tong University School of Medicine, Shanghai, China; 4 Department of Gastroenterology, The Shanghai Tenth People’s Hospital, Tongji University, Shanghai, China; 5 Department of General Surgery, The Fifth People’s Hospital of Wujiang District, Suzhou, China; 6 Center for Inflammatory Bowel Disease Research and Department of Gastroenterology, Affiliated Suzhou Hospital of Nanjing Medical University, Suzhou Municipal Hospital, Gusu School, Nanjing Medical University, Suzhou, China; 7 Cardiac Regeneration and Ageing Lab, Institute of Geriatrics (Shanghai University), Affiliated Nantong Hospital of Shanghai University (The Sixth People’s Hospital of Nantong), Nantong, China; 8 Chongqing Key Laboratory of Precision Optics, Chongqing Institute of East China Normal University, Chongqing, China

**Keywords:** antibiotic, colitis, miRNAs, Occludin, offspring

## Abstract

**Background and aims:**

Antibiotic use in early life is increasingly being scrutinized for its potential effects on health, particularly its association with inflammatory bowel disease (IBD). The weaning period is a critical developmental window for maturation of the intestinal barrier, immune system and gut microbiota. However, it remains unknown how administering antibiotics to male mice starting at weaning affects the susceptibility of their future offspring to IBD.

**Methods:**

Three-week-old weaned male mice were used to investigate the effects of short-term (1 week) and long-term (6 weeks) administration with ampicillin and cefixime on colitis susceptibility in adulthood and in the subsequent F1 generation. Paternal and F1 offspring were administered dextran sodium sulfate (DSS) to induce colitis. Quantitative real-time polymerase chain reaction, immunoblotting, immunofluorescence staining, and histological examination were used to assess mRNA and protein expression and morphological changes. Full 16S rRNA and miRNA sequencing was used to assess changes in the gut microbiota compositions and the mechanisms of intergenerational transmission of intestinal phenotypes. A dual-luciferase reporter assay was used to decipher the direct regulation of miR-10b-5p and miR-200b-3p on Occludin 3′ UTR.

**Results:**

Both short- and long-term antibiotic administration during the weaning period significantly alleviated DSS-induced colitis in male paternal mice, with long-term administration conferring a more pronounced protective effect. Interestingly, the protective effect against DSS-induced colitis was observed in LONG_F1 offspring, which was more pronounced in F1 males than in F1 females. Mechanistically, the protective effect was associated with increased expression of Occludin in epithelial cells, which was negatively modulated by miR-10b-5p and miR-200b-3p. This effect was inherited via the sperm. Furthermore, long-term antibiotic administration significantly altered the gut microbiota of paternal males, increasing the abundance of certain beneficial bacteria, such as *Lactobacillus gasseri* and *Parabacteroides merdae*, while promoting the enrichment of antibiotic-resistant strains.

**Conclusions:**

Long-term antibiotic administration initiated at the weaning stage in male mice improves colitis in adulthood and in their offspring that associates with decreasing miR-10b-5p and miR-200b-3p expression, thus providing a theoretical basis for optimizing the use of antibiotics and prevention of IBD.

## Introduction

Inflammatory bowel disease (IBD) is characterized by chronic and recurrent mucosal inflammation in the gastrointestinal tract. Traditionally, IBD has been considered a disease in high-income countries; however, it is now a global disease with a rapidly increasing incidence worldwide (1). Although awareness of IBD has increased, its overall impact is often underestimated, as it involves intestinal symptoms such as diarrhea and bleeding (2), and affects extra-intestinal organs and whole-body health (3). However, the precise etiologies and pathogenesis of IBD and effective therapeutic strategies remain incompletely elucidated.

Antibiotics are widely used clinical drugs that play an irreplaceable role in the treatment and control of bacterial infections (4, 5). Many previous studies have investigated the profound impact of antibiotic exposure on the gut microbiota diversities and functions, revealing a causal link to the development of IBD (6–8). However, in recent years, the use of antibiotics, especially during early life, has become highly controversial. A large cohort study in Denmark indicated that the incidence of IBD is closely related to the dose, type, and timing of antibiotics use, as well as the age of the patients receiving antibiotics (9). For instance, low-dose penicillin enables precise modulation of susceptible bacteria, exhibiting a protective effect against colitis (10). In the line with these findings, metronidazole, vancomycin, and clindamycin have also been found to significantly reduce the susceptibility of mice to dextran sodium sulfate (DSS)-induced colitis (11). Conversely, antibiotic cocktail-induced depletion of the microbiota suppresses the weaning reaction that essential for the proper immunological maturation, and increases susceptibility to inflammatory diseases in adulthood (12).

Notably, the occurrence of IBD has a familial genetic risk (13, 14). Previous studies have indicated that the gut microbiota can quickly return to its original state after a course of antibiotic treatment. However, emerging studies have confirmed that such disturbances can persist for several months (15, 16). Numerous studies have reported that antibiotic-induced maternal microbial dysbiosis can result in genetically susceptible offspring, thereby increasing the risk of IBD (6, 8, 17). Similarly, paternal antibiotic administration can lead to reproductive system disorders, alter small RNA profiles, and impact the physiology and behavior of the subsequent generation (18, 19).

Intestinal epithelial cells (IECs) are crucial in maintaining gut homeostasis by establishing chemical and physical barriers that prevent the translocation of antigens and pathogens from the intestinal lumen, thereby preventing abnormal immune responses. The weaning reaction is a critical event in the maturation of the intestinal barrier, as demonstrated by research in multiple animal models (20, 21). Furthermore, a complex and tight interplay exist between the intestinal microbiota and host miRNAs. This interaction is crucial in regulating intestinal barrier structure, immune and inflammatory responses, and participates in the onset and progression of IBD (22). Evidently, early-life antibiotic administration can exert lasting and complex effects on the infant gut, altering disease susceptibility in adulthood and potentially impacting the health of subsequent generations. However, the effect of early-life exposure to different antibiotics on IBD susceptibility in adulthood remains unclear, particularly regarding the effects of paternal early-life antibiotic administration on the risk of IBD in offspring, which has not been described previously. The aim of this study was to investigate the effects of early-life antibiotic administration on the intestinal physiology and colitis susceptibility in adults and their offspring.

## Materials and methods

### Animal and treatments

Three-week-old male and 8-week-old female C57BL/6J mice were purchased from Shanghai Laboratory Animal Co., Ltd. (Shanghai, China). All experimental mice were housed in independent ventilation cages under specific-pathogen-free (SPF) conditions. The environmental conditions were maintained at a temperature of 22 ± 3 °C, humidity at 50 ± 10%, and a 12-hour light/dark cycle. All mice had *ad libitum* access to regular diet and water unless otherwise indicated. All animal experiments were conducted in accordance with the guidelines of the Institutional Animal Care and Use Committee of East China Normal University (approval number: m20250602).

In this study, we selected the frequently used antibiotics ampicillin and cefixime, based on the 2022 national outpatient antibiotic usage data published by the National Health Commission of the People’s Republic of China, as the combined antibiotics. Ampicillin and cefixime are the most widely prescribed antibiotics in Chinese clinical practice and are intended to alter, rather than deplete, the intestinal microbiota. After a 3 day acclimation period, the SPF male mice were transferred to a conventional colony facility, and randomly assigned to the control, short-term, and long-term groups according to average body weight (1): control group, which received sterile water containing 0.1% dimethyl sulfoxide (DMSO) for 6 consecutive weeks (designated as CON mice) (2); short-term antibiotic administration group, which received 0.1% DMSO containing 1 g/L ampicillin and 0.24 g/L cefixime for the first week, followed by 0.1% DMSO for the next 5 weeks (designated as SHORT mice) (3); long-term antibiotic administration group, which received 0.1% DMSO containing 1 g/L ampicillin and 0.24 g/L cefixime for 6 consecutive weeks (designated as LONG mice). The dose of antibiotics used was equivalently converted from the human antibiotic dose in accordance with guidance of Food and Drug Administration. (https://www.fda.gov/media/72309/download). After 6 weeks of administration, all three groups of mice were treated with sterile water and recovered for 1 week. Subsequently, these mice were divided into two cohorts. The first cohort of mice were sacrificed and tissues were collected for assessing intestinal healthy status under physiological conditions, while the second cohort of mice used to assess DSS-induced colitis susceptibility.

For establishment of DSS-induced acute colitis, mice received 3% (w/v) DSS (40 kDa, Aladdin; Shanghai, China) in drinking water for 7 days, followed by water for 2 days of recovery. The first day of 3% DSS administration was defined as day 0 (D0). On day 9 (D9), mice were sacrificed and colon tissues were collected for subsequent analyses. Body weight loss and diarrhea scores were recorded daily during the DSS treatment period. Body weight loss was calculated based on the initial body weight of the D0. Diarrhea scores were assessed as described previously (23). Mice were euthanized by CO_2_ inhalation at a flow rate controlled at 9 L/min (60% displacement) for 3 minutes. Euthanasia chambers were transparent 15 L containers with lids and permitted a clear view of each mouse during euthanasia.

### Offspring generation and treatments

The offspring (designated as F1 generation) was generated by natural mating. CON, SHORT, and LONG mice were treated as described above for 7 weeks, and then each male mouse was co-housed with three untreated 8-week-old female mice for 7 days, with all mice kept under standard optimized conditions and allowed *ad libitum* access to regular diet and water. After mating, the males were removed from the cages, and 7–14 days later, the pregnant females were transferred to new cages (one per cage) for parturition. The obtained offspring were designated as Con_F1, Short_F1, and Long_F1, respectively. To assess the impact of paternal antibiotic administration on reproductive outcomes, the number of litters and pups per litter were recorded postpartum. After 3 weeks of birth, male and female pups were separated into different cages. The body weight of the F1 offspring was measured weekly from 3 to 10 weeks old.

At 10 weeks old, Con_F1, Short_F1, and Long_F1 mice (both male and female) were respectively divided into two groups based on average body weight. One group of F1 mice was treated with 3% DSS for 7 days, followed by water for 2 days, and then sacrificed on day 9 (D9) for colon tissue collection. The other group was given water for 9 days and then sacrificed for colon tissue collection.

### Sperm isolation

To collect sperm, the cauda epididymis was dissected from euthanized mice and placed in pre-warmed M2 medium at 37 °C. A needle was used to puncture the cauda epididymis to allow sperm to swim out. After 30 minutes of incubation, sperm were collected by centrifugation at 3000*g* for 5 minutes, washed twice with phosphate buffered saline (PBS), and incubated on ice for 20 minutes in somatic cell lysis buffer (containing 0.1% SDS and 0.5% Triton-100X, diluted with distilled water). Sperm were then collected by centrifugation at 3000*g* for 5 minutes, washed once with PBS, and stored at −80 °C.

### Histological examination and immunofluorescence staining

Distal colon tissues used for histological examination. After being gently rinsed with PBS, segments of the colon were fixed in a 4% paraformaldehyde solution, followed by dehydration in a graded series of ethanol. After dehydration, the specimens were embedded in paraffin. Colonic sections (6-µm thick) were stained with hematoxylin and eosin (H&E, Servicebio; Shanghai, China) performed using standard procedures. An optical microscope (ECLIPSE Ei, Nikon; Tokyo, Japan) and a slide scanning imaging system (SQS-12P, XUYA Scientific; Beijing, China) were used to observe intestinal morphology and acquire images. Histological scores for colonic epithelial damage and inflammatory cell infiltration were performed as described previously (21) in a blinded manner.

For immunofluorescence staining, ileal and colonic segments were fixed in 4% paraformaldehyde solution and transferred to 20% sucrose solution for dehydration overnight. The fixed tissues were subsequently embedded in optimal cutting temperature (OCT) compound and cut into 4-μm sections. Following washing with PBS buffer three times, tissues were permeabilized with PBS buffer containing 0.2% Triton X-100 at room temperature for 15 min and then blocked with PBS buffer containing 5% bovine serum albumin (BSA) at room temperature for 1 h. Sections were incubated with the following primary antibodies including anti-intestinal alkaline phosphatase (Alpi) antibody (1:1500, Servicebio), anti-Mucin 2 (Muc2) antibody (1:800, Servicebio), anti-alpha Defensin 5 (Defa5) antibody (1:300, Servicebio) or anti-Chromogranin A (ChgA) antibody (1:2000, Servicebio) at 4 °C overnight. Alexa Fluor 488 or 555 conjugated secondary antibodies (1:500, Thermo Fisher Scientific) were used at room temperature for 1h and counterstained with DAPI to stain nuclei. Images were acquired using a fluorescence microscope (BX53F2C, Olympus; Tokyo, Japan).

### Quantitative real time-PCR

For gene expression analysis, approximately 50 mg tissue was used to extract total RNA using TRIzol reagent (Invitrogen; Carlsbad, CA, USA) according to the manufacturer’s instructions. The concentration and quality were determined using a NanoDrop 2000 spectrophotometer (Thermo Fisher Scientific) and formaldehyde agarose gel electrophoresis. Complementary DNA (cDNA) was synthesized from 1 μg of total RNA using the Hifair II 1st Strand cDNA Synthesis Kit (Yeasen; Shanghai, China). qRT-PCR analysis was performed using Hieff qPCR SYBR Green Master Mix (Yeasen) on a QuantStudio 5 real-time PCR system (Thermo Fisher Scientific). *Gapdh* was selected as the housekeeping gene. The calculation of mRNA expression levels was analyzed using the 2^−ΔΔCt^ method. The sequences of the primers used is provided in **Supplementary Table 1**.

For bacterial abundance analysis, approximately 50 mg of cecal content was used to extract bacterial genomic DNA using the CTAB/phenol-chloroform method, as previously described (24). Total 16S rRNA gene was used as the housekeeping reference. The relative abundance of *Lactobacillus gasseri* and *Parabacteroides merdae* was calculated using the 2^−ΔΔCt^ method. Primers for 16S rRNA gene were referenced from previous studies: *L. gasseri* (F: 5′-TGGAAACAGRTGCTAATACCG-3′, R: 5′-CAGTTACTACCTCTATCTTTCT -TCACTAC-3′) (25) and *P. merdae* (F: 5′-AGGGTGCGTAGGTGGTGAT-3′, R: 5′-TTCACCGCTACACCACGC-3′) (26).

### Stem-loop qRT-PCR

Stem-loop qRT-PCR was used to detect miRNA expression. As shown in **Supplementary Table 2**, the reverse transcription primers were designed by adding an 8-nt sequence reversed complementary to the 3’ end of the target miRNAs, to the end of the universal stem-loop structure: 5′-CTCAACTGGTGTCGTGGAG -TCGGCAATTCAGTTGAG-3′. Each miRNA required a specific stem-loop primer, and 300 ng of total RNA was reverse transcribed to obtain cDNA. U6 was selected as the housekeeping gene. The qRT-PCR and data analysis were performed using the same methods as described above.

### Western blotting

Proteins from colon tissue were prepared using radio immunoprecipitation assay (RIPA) buffer supplemented with protease and phosphatase inhibitors (Yeasen). Protein concentrations were determined using the BCA protein assay kit (Thermo Fisher Scientific) and immunoblot assay with Zonula occludens 1 (ZO-1, 1:10000, Proteintech; Wuhan, China), Occludin (Ocln, 1:10000, Proteintech), Claudin (Cldn, 1:5000, Proteintech), and HSP90 (1:10000, Proteintech) antibodies. The specific proteins were visualized using BeyoECL Moon (Beyotime; Shanghai, China) and captured on a chemiluminescence analysis system Tanon 5200 (Tanon; Beijing, China). The band intensities were quantitated using Quantity One (Bio-Rad Laboratories) and normalized to that of HSP90.

### Cell culture and treatments

CCD841 and 293T cell lines were originally obtained from the American Type Culture Collection (ATCC; Rockville, Maryland, USA). All cells were cultured and maintained in Dulbecco’s modified Eagle’s medium (high glucose, 4.5 g/L) supplemented with 10% fetal bovine serum, 50 μg/mL streptomycin, and 50 U/mL penicillin at 37 °C with 5% CO_2_ according to the ATCC instructions. For miRNA interference experiments, CCD841 cells were cultured in 12-well plates and transfected with miR-10b-5p mimics, miR-200b-3p mimics or negative control (NC) using Lipofectamine 3000 (Invitrogen) at 50–70% confluency, according to the manufacturer’s instructions. The sequences of miRNA mimics used is provided in **Supplementary Table 3**.

### Plasmid construction and dual luciferase reporter assay

The wild-type Ocln 3′ untranslated region (UTR) sequence, which contains the binding sites for miR-10b-5p and miR-200b-3p, was obtained by PCR amplification using mouse colonic cDNA as a template, with primers designed by querying NCBI. The sequence was cloned downstream of the Firefly luciferase gene in the pmirGLO vector (Promega; Madison, Wisconsin, USA) using double restriction enzyme digestion. Mutation primers were designed based on the seed sequences of the miR-10b-5p and miR-200b-3p binding sites on the Ocln 3′ UTR, and site-directed mutagenesis kits (Yeasen) were used to construct pmirGLO plasmids containing mutated Ocln 3′ UTR sequences (3′ UTR-m10b and 3′ UTR-m200b). All vectors were confirmed by sequencing at Biosune Biotechnology Co., LTD (Shanghai, China). The sequences of the primers used for vector construction is provided in **Supplementary Table 4**.

For dual luciferase reporter assay, 293T cells were co-transfected with pmirGLO plasmids containing the inserted sequences and either miR-10b-5p mimics, miR-200b-3p mimics, or NC mimics using Lipofectamine 2000 (Invitrogen). After 48 hours of incubation, Firefly luciferase and Renilla luciferase activities were measured using a dual-luciferase reporter assay kit (Yeasen). Firefly luciferase activity was normalized to Renilla luciferase activity for each sample, and the resulting ratios were further normalized to the NC group to determine the relative luciferase activity.

### Small RNA sequencing and data analysis

Total RNA from colonic tissues was extracted using the methods as described above. Only high-quality RNA sample (OD260/280 = 1.8~2.2, OD260/230≥2.0, RQN≥7, 28S:18S≥1.0, >1 μg) was used to construct sequencing library. A total amount of 1 μg total RNA per sample was used as input material for the small RNA library. Sequencing libraries were generated using QIAseq miRNA Library Kit (Qiagen) following manufacturer’s recommendations. The activated 5′ and 3′ adaptors were ligated to the total RNA, respectively. Then the adaptor-ligated RNA was transcribed into first-strand cDNA by using reverse transcriptase and random primer. A PCR reaction was performed using primers complementary for 11–12 cycles, fragments of appropriate size were isolated by a 6% Novex TBE PAGE gel. After quantified by Qubit 4.0, single-end RNA-seq sequencing library was sequenced with the Illumina NovaSeq Xplus sequencer.

The mapped small RNA tags were used to identifying known miRNA with miRBase 22.0 database (http://www.mirbase.org/) as reference. The expression level of each miRNA was calculated according to the transcripts per million reads (TPM) method. Differential expression analysis was performed using the DESeq2 (27). Differential expressed genes (DEGs) with |FC|≥1.2 and FDR<0.05 were considered to be significantly different expressed genes. The target genes of miRNAs were predicted using miRWalk and miRTarBase databases. The accession number for the sequencing data reported in this paper is SRA: PRJNA1303027.

### 16S rRNA sequencing and data analysis

Fresh fecal samples were collected, immediately frozen and stored. Genomic DNA from the feces was extracted using the cetyltrimethylammonium bromide method as described previously (24). After obtaining total fecal DNA, the bacterial 16S rRNA genes were amplified using the universal bacterial primers 27F (5’-AGRGTTYGATYMTGGCTCAG-3’) and 1492R (5’-RGYTACCTTGTT -ACGACTT-3’) (28), with the PCR process being replicated three times. Primers were tailed with PacBio barcode sequences to distinguish each sample. Following this, The PCR products were purified using the AMPure^®^ PB beads (Pacifc Biosciences; Menlo Park, CA, USA) and quantified with Qubit 4.0 (Thermo Fisher Scientific). Purified products were pooled in equimolar and DNA library was constructed using the SMRTbell prep kit 3.0 (Pacifc Biosciences) according to PacBio’s instructions. Purified SMRTbell libraries were sequenced on the Pacbio Sequel IIe System (Pacifc Biosciences). High-fidelity reads were obtained from the subreads, generated using circular consensus sequencing via SMRT Link v11.0.

Alpha diversity metrics at the amplicon sequence variants (ASVs) level based on Shannon’s index were calculated with Mothur v1.30.1 (http://www.mothur.org/wiki/Calculators) (29). The similarity among the microbial communities in different samples was determined by principal coordinate analysis (PCoA) based on Bray–curtis dissimilarity using Vegan v2.5–3 package. Analyses of alpha diversity, beta diversity, and generation of bacterial composition bar charts and heatmaps were performed at Majorbio Cloud platform (https://cloud.majorbio.com). The accession number for the sequencing data reported in this paper is SRA: PRJNA1303393.

### Measurement of antibiotic-resistant strains

The parental stools were collected at D0, 100 mg stool samples were homogenized in 1 mL sterile anaerobic PBS, filtered and diluted 10^2^-fold. Then, 100 uL dilution was added to brain heart infusion (BHI) agar plates (containing 100 μg/mL ampicillin and 25 μg/mL cefixime) and cultured aerobically or anaerobically at 37 °C for 48 h. The bacterial 16S rRNA gene was amplified from colonies by PCR using the universal primers 27F: 5’-AGRGTTYGA-TYMTGGCTCAG-3’ and 1492R: 5’-RGYTACCTTGTTACG -ACTT-3’. The resulting amplicons were purified from agarose gel and subsequently sequenced using the 27F primer. The identity of the antibiotic-resistant strains was determined by aligning the sequencing results to the NCBI database using Nucleotide BLAST (https://blast.ncbi.nlm.nih.gov/Blast.cgi).

### Statistical analysis

Data are presented as the mean ± standard error of the mean (SEM). Graphpad Prism 9 (version 9.5.1, GraphPad Software; San Diego, CA, USA) was used for statistical analysis. The statistical significance of the differences was tested using unpaired two-tailed Student’s *t* test, Wilcoxon rank-sum test, one-way ANOVA or two-way ANOVA followed by Tukey’s *post hoc* test. Spearman’s correlation analysis was performed to analyze the correlation between miRNAs and Ocln expression. The results were considered as statistically significant at *P* < 0.05.

## Results

### Antibiotic administration in weaned male mice improves intestinal epithelial functions under physiological conditions in adulthood

**Figure 1:**
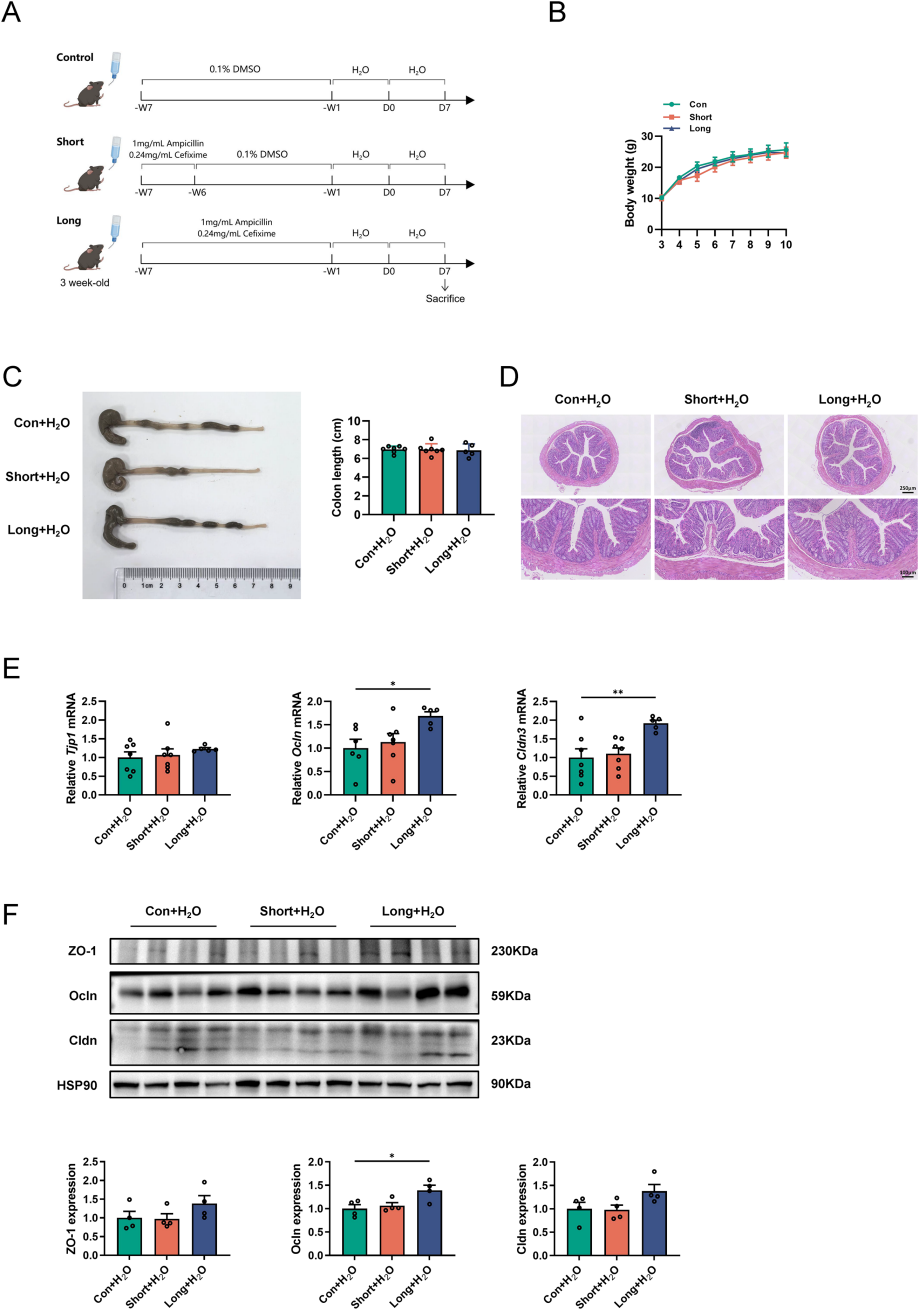
Antibiotic administration in weaned male mice improves intestinal epithelial function in adulthood under physiological conditions **(A)** Schematic diagram for the mouse model of antibiotic-treated weaned male mice in physiological condition. n=5–7 mice per group. **(B)** Body weight were measured every week since 3-week-old. **(C, D)** Colon length and H&E staining in each group. n=5–7 mice per group. Scale bar, 100 μm (down) and 250 μm (upper). **(E)** Relative mRNA levels of *Tjp1*, *Ocln*, and *Cldn3* in colon were evaluated by qRT-PCR. **(F)** Protein levels of ZO-1, Ocln, and Cldn in colon were evaluated by Western blot. Quantitative analysis of the results was performed using Image J Data are expressed as the mean ± SEM; Statistical significance was determined using one-way ANOVA followed by Tukey’s multiple comparisons test; *P < 0.05, **P < 0.01 and ***P < 0.001.

To investigate the effect of antibiotic administration in early life on intestinal physiological changes in adulthood, 3-week-old weaned male mice were randomly divided into three groups, and the procedures of treatment as shown in **Figure 1A**. Body weight, colon length, and histological morphology did not differ significantly among the three groups (**Figures 1B–D**). The expression of genes related to the intestinal barrier (e.g., *Ocln* and *Cldn3*) was significantly upregulated in the colons of LONG mice compared to that in CON mice (**Figure 1E**). Notably, only the Ocln protein expression increased significantly in the colon of LONG mice (**Figure 1F**). We further assessed the effects of antibiotic administration on intestinal epithelial function. The intestinal epithelium mainly consists of enterocytes, goblet cells, Paneth cells, and enteroendocrine cells, which play key roles in the intestinal barrier and innate immunity (30). The mRNA level of the goblet cell marker (*Muc2*) was significantly increased, whereas *Chga*, an enteroendocrine cell marker in the ileum of SHORT and LONG mice was lower than in CON mice (**Supplementary Figure 1A**). Immunofluorescence staining confirmed the upregulation of Muc2 in the ileum of LONG mice. In contrast, the canonical marker Alpi for enterocytes, Defa5 for Paneth cells, or ChgA for enteroendocrine cells did not alter (**Supplementary Figures 1B–E**). Both gene and protein expression of *Muc2* in the colon were consistently significantly higher in LONG mice than in CON mice (**Supplementary Figures 1F, G**). These results indicate that both short- and long-term antibiotic administration during the weaning period of male mice improves intestinal epithelial phenotypes under physiological conditions.

### Antibiotic administration in weaned male mice improves DSS-induced colitis in adulthood

**Figure 2:**
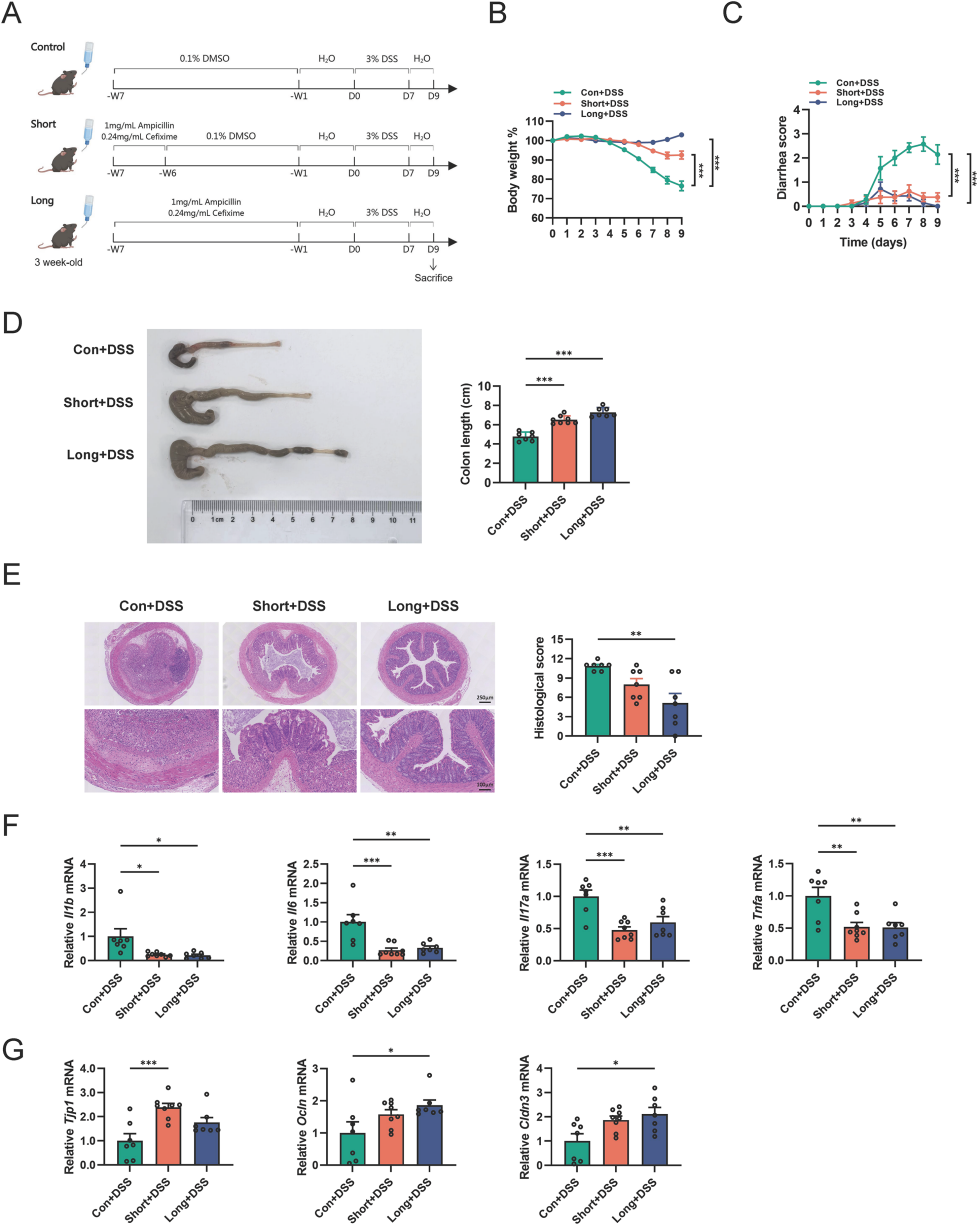
Antibiotic administration in weaned male mice improves DSS-induced colitis in adulthood **(A)** Schematic diagram for the mouse model of antibiotic-treated weaned male mice and DSS-induced colitis. Antibiotics including 1 g/L ampicillin and 0.24 g/L cefixime dissolved in drinking water were provided for the specified duration. n=7–8 mice per group. **(B, C)** Body weight and diarrhea scores were examined every day during the course of DSS treatment. **(D)** Gross morphology and length of the colon in different groups. **(E)** Colonic H&E staining after DSS challenge. The right panel is the histological scores. Scale bar, 100 μm (down) and 250 μm (upper). **(F)** Relative mRNA levels of *Il1b, Il6, Il17a* and *Tnfa* in the distal colon were evaluated by qRT-PCR. **(G)** Relative mRNA levels of *Tjp1*, *Ocln* and *Cldn3* in the distal colon were evaluated by qRT-PCR. Data are expressed as the mean ± SEM. Statistical significance was determined using one-way or two-way ANOVA followed by Tukey’s multiple comparisons test; *P < 0.05, **P < 0.01 and ***P < 0.001.

We next investigated the impact of antibiotic administration during the weaning period on acute colitis in adulthood by challenging mice with DSS, a chemical irritant that induces intestinal inflammation exhibiting clinical and histological features of ulcerative colitis (31). The procedures of treatment as shown in **Figure 2A**. Upon DSS administration, the clinical signs of colitis, including body weight loss (from Day 5 onward), rectal bleeding (from Day 5 onward), and colon shortening, were significantly alleviated in both SHORT and LONG mice compared to those in CON mice (**Figures 2B–D**). H&E staining showed that the histological scores of LONG mice were significantly lower than those of CON mice, characterized by a greater number of colonic epithelial goblet cells, more intact crypt architecture, and less mucosal inflammatory infiltration (**Figure 2E**). Accordingly, the mRNA levels of pro-inflammatory cytokines [e.g., *Interleukin 1 beta* (*Il1b)*, *Il6*, *Il17a* and *tumor necrosis factor alpha* (*Tnfa)*] were decreased in the colons of SHORT and LONG mice compared to those in CON mice (**Figure 2F**). In addition, the mRNA levels of intestinal barrier-related genes [e.g., *tight junction protein 1* (*Tjp1)*, *Ocln*, and *Cldn3*] in the colon were significantly increased in LONG mice compared to those in CON mice (**Figure 2G**). Hence, these data indicate that antibiotic administration in weaned male mice significantly alleviates DSS-induced colitis in adulthood, exhibiting a more pronounced protective effect with long-term antibiotic administration.

### Antibiotic administration in weaned male mice increases the gene expression of the intestinal barrier under physiological conditions in the offspring

To investigate intestinal physiology and colitis susceptibility in the offspring of antibiotic-treated male mice, we co-housed male mice with untreated 8-week-old females to generate F1 offspring (CON_F1, SHORT_F1, and LONG_F1 mice) (**Supplementary Figure 2A**). There were no significant differences in the number of pups per litter or pregnancy rates among the groups (**Supplementary Figure 2B, C**), indicating that antibiotic administration did not significantly affect male fertility. All offspring grew normally and their body weights did not differ at 10 weeks of age among the groups (**Supplementary Figure 2D**).

We then analyzed the intestinal health of the F1 offspring under physiological conditions. For the F1 male offspring, the colon length and histological morphology did not differ significantly among the three groups (**Supplementary Figures 3A, B**). Interestingly, the mRNA and protein expression levels of *Tjp1* and *Ocln* were significantly increased in LONG_F1 mice compared to CON_F1 mice (**Supplementary Figures 3C, D**). However, the marker gene expression of intestinal epithelial function showed no marked changes among the three groups (**Supplementary Figure 4A**). Immunofluorescence staining further confirmed no significant differences in the expression of Alpi, Muc2, Defa5, and Chga in the ileum or Muc2 in the colon (**Supplementary Figures 4B–G**), indicating that the number and function of IECs in F1 male offspring are not significantly altered. Similar results were observed in the F1 female offspring under physiological conditions (**Supplementary Figures 5**, **S6**). Overall, these results indicate that antibiotic administration in weaned male mice increases the gene expression of the intestinal barrier but does not significantly affect the intestinal epithelial functions of F1 offspring under physiological conditions.

### Antibiotic administration in weaned male mice improves DSS-induced colitis in the offspring

**Figure 3:**
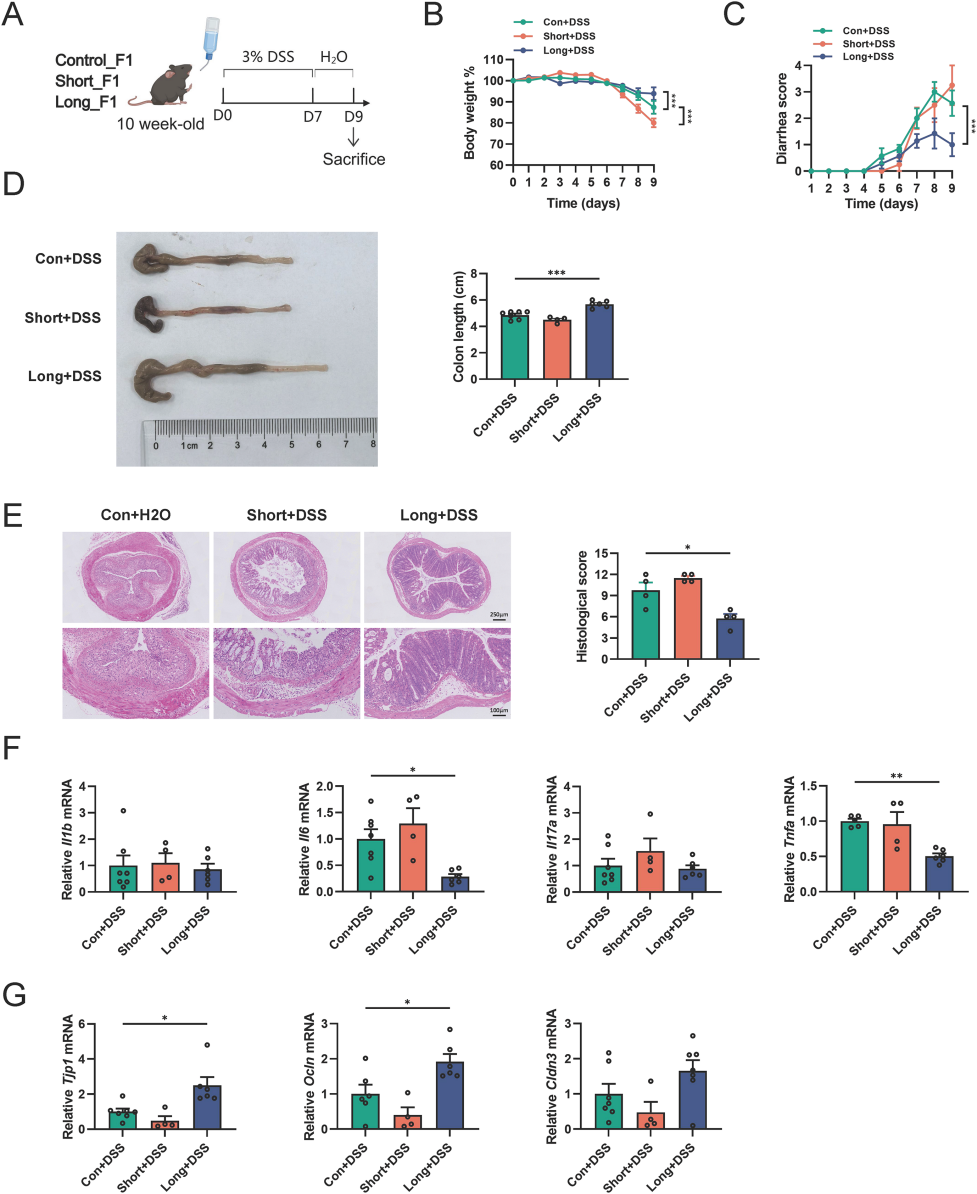
Antibiotic administration in weaned male mice improves DSS-induced colitis in the offspring **(A)** Schematic diagram for the mouse model of DSS-induced colitis in F1 male offspring. n=4–7 mice per group. **(B, C)** Body weight and diarrhea scores of F1 male mice were examined every day during the course of DSS treatment. **(D)** Gross morphology and length of the colon in different groups. **(E)** Colonic H&E staining after DSS challenge. The right panel is the histological scores. Scale bar, 100 μm (down) and 250 μm (upper). **(F)** Relative mRNA levels of *Il1b, Il6, Il17a*, and *Tnfa* in the distal colon were evaluated by qRT-PCR. **(G)** Relative mRNA levels of *Tjp1*, *Ocln*, and *Cldn3* in the distal colon were evaluated by qRT-PCR. Data are expressed as the mean ± SEM. Statistical significance was determined using one-way or two-way ANOVA followed by Tukey’s multiple comparisons test; *P < 0.05, **P < 0.01 and ***P < 0.001.

Subsequently, the offspring were administered 3% DSS in drinking water for 7 days to induce colitis (**Figure 3A**). Notably, LONG_F1 male mice exhibited an alleviated severity of colitis compared to controls, including lower body weight loss (at Day 9), diarrhea scores (from Day 7 onward), and colon shortening (**Figures 3B–D**). Histological analysis of the colonic sections revealed that mucosal damage, including mucosal erosion, crypt and goblet cell loss, and lymphocyte infiltration, was mitigated in LONG_F1 male mice (**Figure 3E**), and the mRNA levels of pro-inflammatory cytokines (e.g., *Il6* and *Tnfa*) in the colons of LONG_F1 male mice were significantly lower than those in the control group (**Figure 3F**). The expression of intestinal barrier-related genes (e.g., *Tjp1* and *Ocln*) in the colon was significantly increased in LONG_F1 male mice (**Figure 3G**). However, no protective effect against DSS-induced colitis was observed in SHORT_F1 male mice (**Figures 3B–G**).

Similarly, we administered 3% DSS to the F1 female offspring to induce colitis. The results showed that LONG_F1 female mice had lower body weight loss, diarrhea scores, and colon shortening than those in the other groups, indicating mild alleviation of colitis (**Supplementary Figures 7A–C**). However, there were no significant differences in histological scores and gene expression of pro-inflammatory cytokines or intestinal barrier between LONG_F1 and CON_F1 female mice (**Supplementary Figures 7D–F**). Conversely, SHORT_F1 female mice exhibited a more severe colitis phenotype, with significantly greater body weight loss and diarrhea scores; however, the colon shortening, histological scores, and gene expression of pro-inflammatory cytokines or intestinal barrier showed no marked changes (**Supplementary Figures 7A–F**). Therefore, long-term antibiotic administration in weaned male mice improves DSS-induced colitis in adulthood and in their male offspring, suggesting that the phenotype displays intergenerational transmission, which is more evident in the F1 male generation.

### Decreased miR-10b-5p and miR-200b-3p expression contributes to upregulation of Ocln in LONG_F1 mice

**Figure 4:**
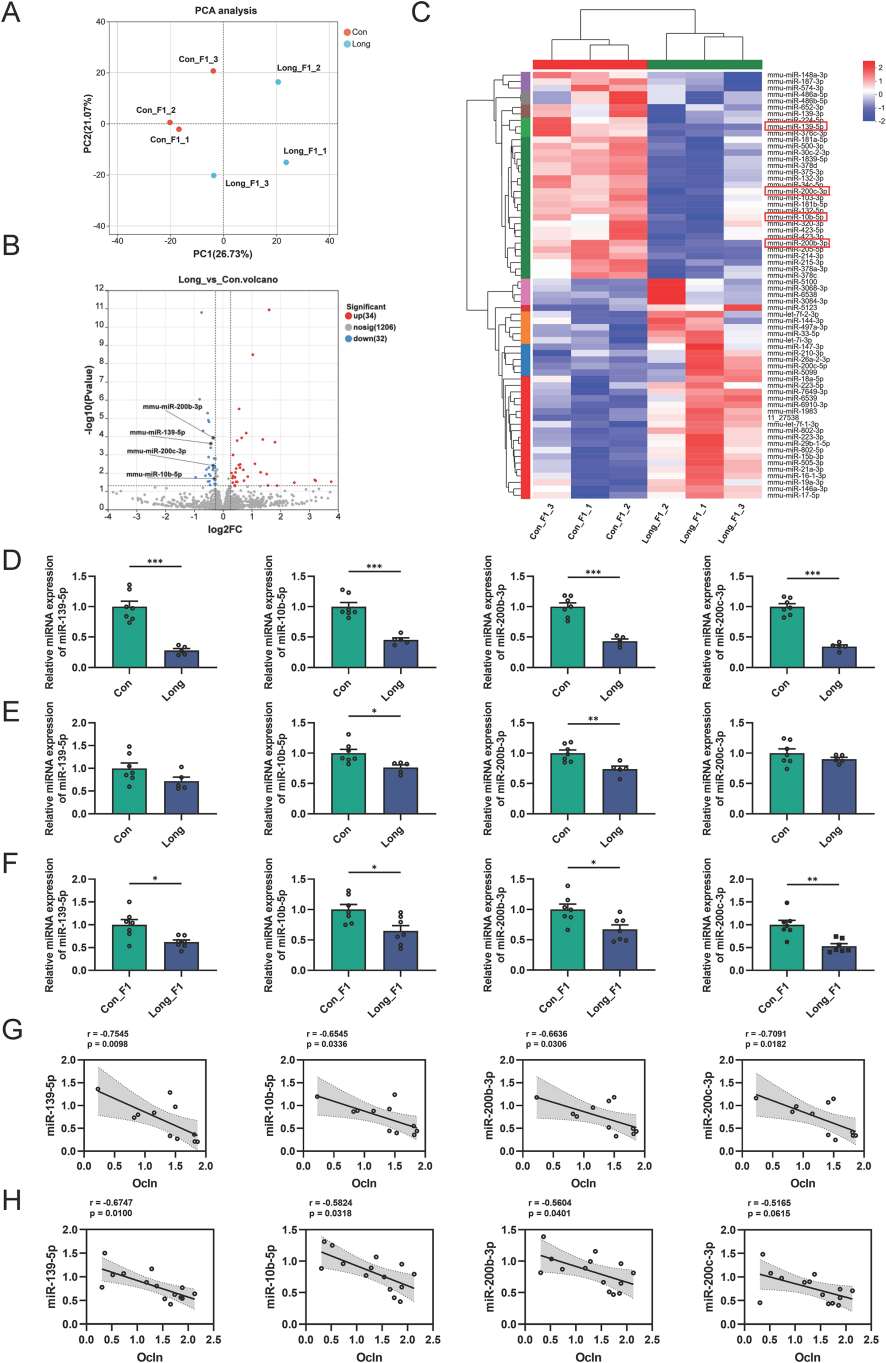
miRNA transgenerational transmission in the colon of LONG_F1 male mice **(A)** Principal component analysis (PCA) analysis of small RNAs in the colon from physiological CON_F1 and LONG_F1 male mice. **(B)** Volcano plot of miRNA expression level differences between CON_F1 and LONG_F1 male mice in colon. **(C)** Heatmap of differentially expressed miRNAs in colon of CON_F1 and LONG_F1 male mice. miRNAs highlighted with red boxes were predicted to bind to the Ocln 3’ UTR. **(D-F)** Relative levels of miR-139-5p, miR-10b-5p, miR-200b-3p and miR-200c-3p in paternal colon **(D)**, paternal sperm **(E)** and the colon of F1 male mice **(F)** were evaluated by qRT-PCR. n=5–7 mice per group. **(G, H)** Spearman correlation analysis between miRNAs expression and Ocln expression in the colon of parental and F1 male mice under physiological conditions, respectively. Data are expressed as the mean ± SEM. Statistical significance was determined using unpaired two-tailed t-test; *P < 0.05, **P < 0.01 and ***P < 0.001.

Integrating the intestinal phenotypes of the paternal and F1 generations under physiological and inflammatory conditions, it is apparent that increased gene expression of the intestinal barrier (e.g., *Tjp1* or *Ocln*) may improve colitis susceptibility in long-term antibiotic treated-male mice and their offspring. Previous studies have shown that the paternal gut microbiota is not inherited by the offspring via intergenerational inheritance, whereas phenotypes can be passed on to the offspring via sperm-mediated epigenetic inheritance (18). Epigenetics typically includes DNA methylation, histone modifications, and non-coding RNAs (32). DNA methylation and histone modifications undergo extensive reprogramming during early embryonic development, posing challenges to their transgenerational stability. However, a growing body of evidence suggests that sperm-derived small RNAs exhibit a certain resistance to reprogramming and can serve as carriers of epigenetic information, influencing gene expression and phenotypes in offspring (33, 34). Therefore, we hypothesized that the phenotype of LONG_F1 male mice may be inherited specifically via miRNAs in paternal gametes. We then performed small RNA sequencing analysis of the colon from CON_F1 and LONG_F1 male mice. Principal component analysis (PCA) of the miRNA profiles revealed a clear separation between CON_F1 and LONG_F1 male mice (**Figure 4A**). Next, we identified 66 miRNAs that differed between the two groups, in which 34 increased and 32 significantly reduced (**Figure 4B**). The enrichment analysis of targets of up-expressed miRNAs showed marked enrichment in signaling pathways regulating pluripotency of stem cells, axon regeneration, and pathways in cancer, whereas targets of down-expressed miRNAs enriched in FoxO signaling pathway, neurotrophin signaling pathway, and human papillomavirus infection (**Supplementary Figures 8A, B**). Among the differential expressed miRNAs, we focused on 4 miRNAs that target Ocln as predicted, including miR-139-5p, miR-10b-5p, miR-200b-3p, and miR-200c-3p (**Figures 4B, C**). We measured the expression of these four miRNAs in the colon and sperm of fathers from the CON and LONG groups, as well as in the colon of F1 male mice to verify whether they were inherited from the father to the offspring via the sperm. The results showed that only miR-10b-5p and miR-200b-3p expression levels in LONG mice significantly decreased in all tissues (**Figures 4D, F**). Moreover, these two miRNAs were significantly negatively correlated with Ocln expression in the colons of parental and F1 male mice (**Figures 4G, H**). These findings suggest that the changes in miR-10b-5p and miR-200b-3p expression in the colon of F1 male mice are indeed transmitted from the father via sperm.

**Figure 5:**
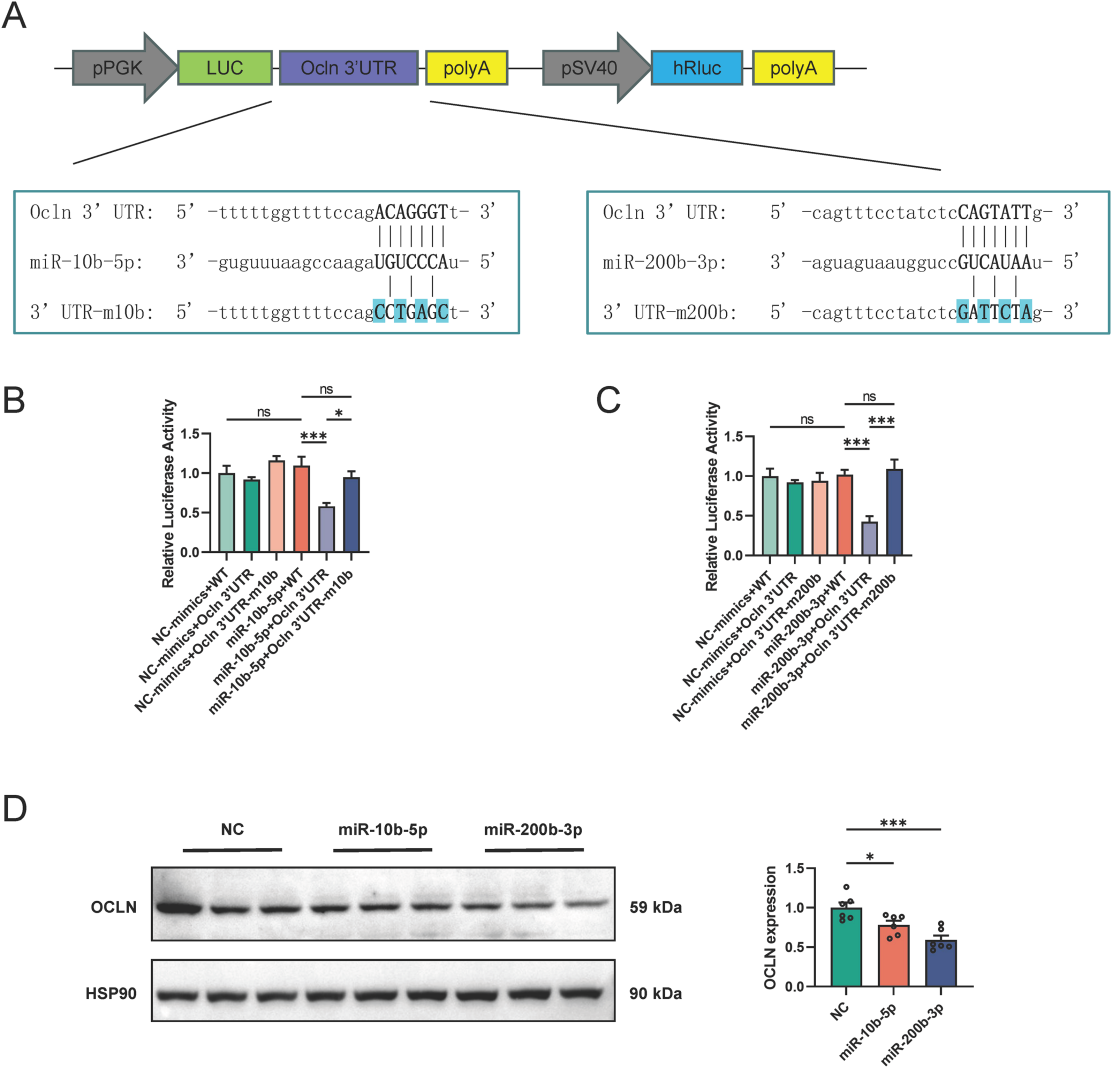
Regulation of Ocln expression by miR-10b-5p and miR-200b-3p **(A)** Construction of the dual-luciferase reporter plasmids and schematic diagram of the predicted miR-10b-5p and miR-200b-3p binding sites on the 3’ UTR of Ocln in mice. **(B, C)** Dual-luciferase experiments were performed to examine the binding efficiency of miR-10b-5p or miR-200b-3p to Ocln 3′ UTR in 293T cells. 293T transfected with wild-type or mutated Ocln 3′ UTR luciferase constructs and negative control (NC) mimics, miR-10b-5p mimics or miR-200b-3p mimics. n=6 for each group. **(D)** The expression level of Ocln was evaluated by WB after transfection of miR-10b-5p mimics or miR-200b-3p mimics for 48 hours in CCD841 cells. Quantitative analysis of the results was performed using Image J. n=6 for each group. Data are expressed as the mean ± SEM. Statistical significance was determined using one-way ANOVA followed by Tukey’s multiple comparisons test; *P < 0.05, **P < 0.01 and ***P < 0.001.

To determine whether miR-10b-5p and miR-200b-3p are directly involved in the regulation of Ocln expression, the binding sites of these miRNAs on the Ocln 3′ UTR were predicted using the miRanda software. Subsequently, we constructed dual-luciferase reporter vectors containing either the wild-type Ocln 3′ UTR or mutated 3′ UTR (3′ UTR-m10b and 3′ UTR-m200b) (**Figure 5A**). Next, miR-10b-5p and miR-200b-3p mimics were co-transfected with the vectors into 293T cells to overexpress the miRNAs. As expected, miR-10b-5p and miR-200b-3p mimics significantly reduced the activity of the Ocln 3′ UTR, whereas mutations in the binding motifs for miR-10b-5p and miR-200b-3p abolished the effects of these miRNAs on luciferase activity (**Figures 5B, C**). These findings suggest that miR-10b-5p and miR-200b-3p bind directly to the Ocln 3′ UTR, thereby regulating Ocln expression. Accordingly, the Ocln protein levels were significantly reduced when miR-10b-5p and miR-200b-3p mimics were transfected into the CCD841 cells (**Figure 5D**). Hence, increased Ocln expression in the colons of LONG mice was inherited by LONG_F1 male mice via modulating sperm miR-10b-5p and miR-200b-3p expression, which conferred these mice with a colitis-resistant phenotype.

### Long-term antibiotic administration in weaned male mice alters gut microbiota compositions in adulthood

**Figure 6:**
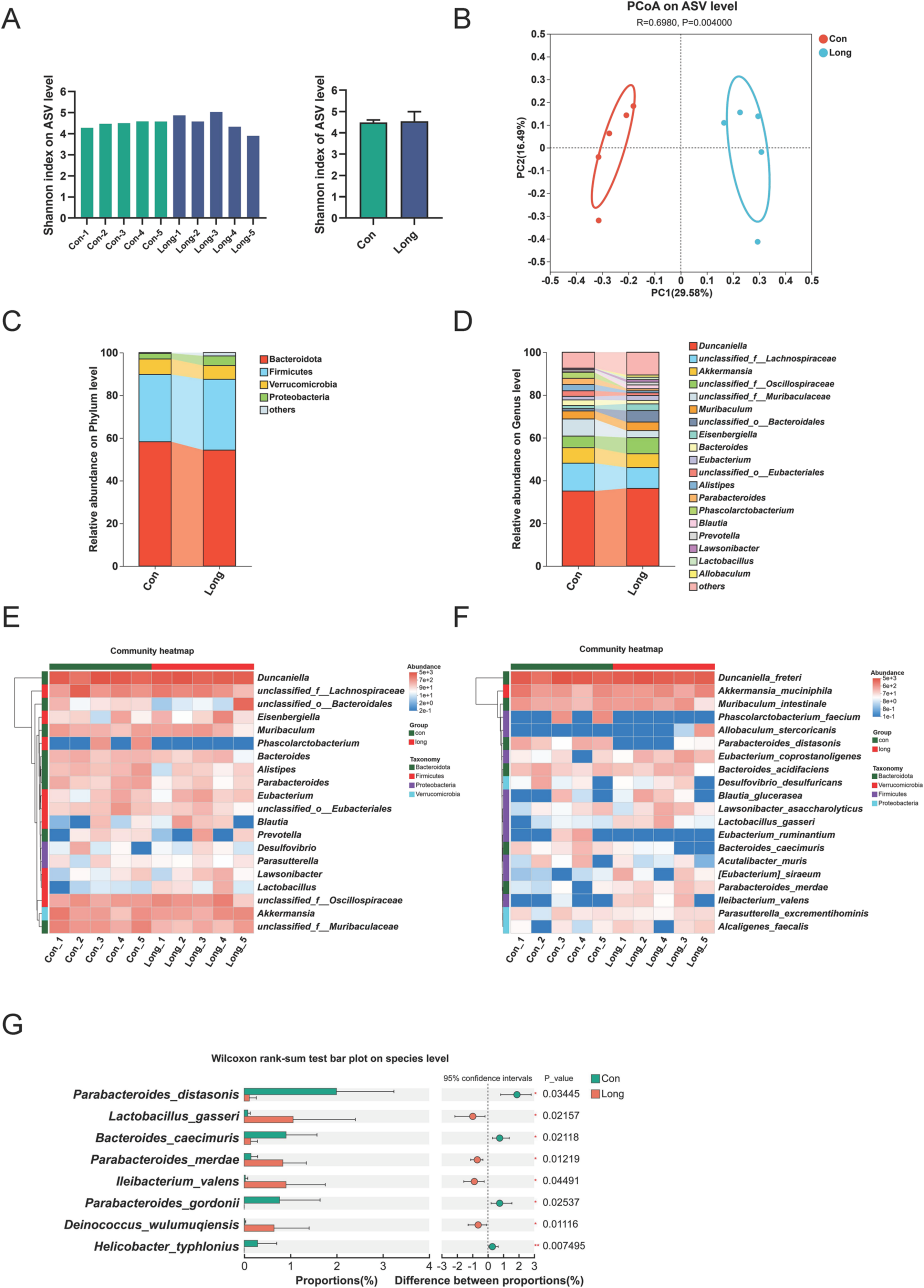
Long-term antibiotic administration in weaned male mice alters gut microbiota compositions in adulthood Stool samples were collected from CON and LONG paternal male mice two weeks after the end of antibiotic administration for full-length 16S rRNA sequencing analysis. n=5 mice per group. **(A)** Bacterial alpha diversity based on Shannon index. **(B)** Principal coordinate analysis (PCoA) of gut microbiota in CON and LONG groups based on Bray-Curtis distances. **(C, D)** The mean relative abundance at phylum **(C)** and genus **(D)** level of bacteria in CON and LONG groups. The bars list the top 20 genera in abundance proportion. **(E, F)** The relative abundance of bacteria at genus **(E)** and species **(F)** level in CON and LONG groups. These heatmaps were composed of the top 20 genera or species in abundance. **(G)** Bar chart showing the 8 species with the greatest difference in average relative abundance between the CON and LONG groups. Data are expressed as the mean ± SEM. Statistical significance was determined using Wilcoxon rank-sum test; *P < 0.05 and **P < 0.01.

Although we focused on miRNA-mediated intergenerational transmission in the intestinal barrier in this study, the effects of antibiotic administration on gut microbiota were also evaluated using full-length bacterial 16S rRNA sequencing. After 2 weeks of recovery, α-diversity, as measured using the Shannon index, did not differ significantly between CON and LONG male mice (**Figure 6A**). PCoA based on the Bray–Curtis distance confirmed that the gut microbial composition of LONG mice was distinct from that of CON mice, which was consistent with bacterial composition analysis at the phylum and genus levels (**Figures 6B, D**). A heatmap was constructed with the top 20 most abundant genera, showing that the abundance of *Lactobacillus*, *Blautia*, *Lawsonibacter*, and *Eisenbergiella* increased, while that of *Bacteroides*, *Parabacteroides*, and *Alistipes* decreased in the LONG group (**Figure 6E**). A detailed analysis of the bacterial species illustrated that long-term antibiotic administration increased the abundance of *Lactobacillus gasseri*, *Parabacteroides merdae*, *Ileibacterium valens*, *Deinococcus wulumuqiensis*, and *Blautia glucerasea*, whereas it decreased the abundance of *Parabacteroides distasonis*, and *Bacteroides caecimuris* compared to those in the controls (**Figures 6F, G**). Previous studies have reported that the bacteria *L. gasseri (*35*)*, *P. merdae (*36*)*, and *D. wulumuqiensis (*37) could alleviate colitis and resist oxidative stress, suggesting that decreased colitis susceptibility due to long-term antibiotic administration may be associated with gut microbial alterations.

To further investigate the role of microbiota changes in the paternal or offspring intestines. We selected two probiotics, *L. gasseri* and *P. merdae*, which were highly elevated in the LONG group, and performed correlation analyses between their abundance with the levels of miR-10b-5p and miR-200b-3p in the paternal colon, respectively. The results showed that abundance of these two probiotics was significantly negative correlated with levels of the miRNAs (**Supplementary Figure 9A**). However, no significant changes in the abundance of *L. gasseri* and *P. merdae* were observed in the intestine of F1 offspring, except for the reduced abundance of *P. merdae* in LONG_F1 female mice (**Supplementary Figure 9B, C**). Moreover, there was no significant correlation between probiotic abundance and colonic miR-10b-5p or miR-200b-3p levels in the intestine of F1 male mice (**Supplementary Figure 9D**). These findings suggest that antibiotic-induced alterations in the paternal gut microbiota likely contribute to modulating paternal intestinal miRNA levels, whlie can not be directly inherited to shape the offspring’s gut phenotype.

Considering that antibiotic administration induces the formation of drug-resistant strains (38), we further estimated the abundance of resistant strains using a plate culture test and then plated serial dilutions of stool suspensions from Long and CON mice onto BHI agar supplemented with ampicillin and cefixime. The results showed that the number of ampicillin and cefixime-resistant bacteria in the stool of LONG mice significantly increased (**Supplementary Figure 10A**). Clony PCR and BLAST assays further determined that these drug-resistant strains are mainly *Enterobacter*, *Alcaligenes* and *Enterococcus* (**Supplementary Figure 10B**). These findings indicate that long-term antibiotic administration increases the abundance of antibiotic-resistant strains, representing a potential safety risk to the intestinal environment.

## Discussion

The pathogenesis of IBD is complex, and the underlying mechanism of which involves genetics, immunity, microbiota, and various omics (39), is influenced by multiple factors such as drugs, environment, diet, and stress (40). Antibiotics can directly impact the composition of the gut microbiota, as well as influence immunity and gut health, are all closely related to the occurrence of IBD (6, 41). In particular, the effects of antibiotics on the gut microbiota are transmitted from pregnant dams to newborn pups via the birth canal, resulting in abnormal adaptive immunity and increased susceptibility to colitis in the offspring (8, 42). However, the impact of paternal antibiotic administration on IBD susceptibility in offspring remains unclear.

The weaning reaction is a critical event for the intestinal barrier and immune maturation, with long-lasting effects on future intestinal health (20). Emerging studies have shown that the use of antibiotics during the weaning reaction period disrupts the intestinal barrier and intestinal immunity, and induces dysregulation of gut microbiota, thereby increasing the risk of colitis (43, 44). In contrast, our results showed that both short- and long-term antibiotic administration in 3-week-old weaned male mice conferred protection against DSS-induced colitis in adulthood. Similar findings have been reported in previous studies (10, 45). Due to differences in the duration, quantity, and antimicrobial spectrum of antibiotics used, inconsistent outcomes are often reported (46). It is noteworthy that previous studies aimed at comprehensive and sustained depletion of the microbiota by using an antibiotic cocktail, thereby inhibiting the weaning reaction (12, 46). In contrast, in this study, we administered ampicillin and cefixime to weaned male mice merely to interfere with the composition of the gut microbiota, without aiming for its depletion. Antibiotics have direct impacts on mammalian cells, including the induction of mitochondrial dysfunction, oxidative damage, or compromise of the mucosal barrier (47). However, this is inconsistent with the intestinal barrier protection effects that observed in this study. Therefore, we speculate that this protective effect is not a direct effect of antibiotics on intestinal epithelial cells, while an indirect effect after the remodeling of microbial community structure.

We generated F1 mice and examined whether the protective effect against DSS-induced colitis in antibiotic-treated parental mice was transmitted to the offspring. Notably, the results showed that the F1 offspring of the long-term group exhibited protection against DSS-induced colitis. We analyzed phenotypic changes in the intestinal barrier and epithelial function of fathers and offspring under physiological or DSS-induced conditions to explain the mechanism underlying this protective transmission. Both gene and protein levels of Ocln in the colon were significantly increased in the fathers of the LONG group and their F1 male mice. Ocln is an integral membrane protein involved in the formation of intestinal tight junctions and plays a crucial role in regulating the mucosal barrier and intestinal inflammation (48). Studies have shown that the expression of Ocln is decreased significantly and intestinal permeability is reduced when solid food intake begins during the weaning period (20, 49). Thus, tight junction proteins play an important role in intestinal maturation, and early-life salutary antibiotic administration may accelerate this process (50). Therefore, we hypothesized that the protection against colitis in antibiotic-treated weaned fathers, as well as in their offspring, may be due to the elevated expression of Ocln.

Intergenerational inheritance of this phenotype suggests an epigenetic mechanism. Previous studies have shown that epididymal somatic cells can actively transport self-synthesized or packaged sncRNAs (including miRNAs) to sperm via secreted extracellular vesicles (51–53), which serve as important epigenetic mediators to transmit father information to offspring and to influence the gene expression of offspring (54). Therefore, we considered that the miRNAs present in sperm may play a key role in the intergenerational regulation of Ocln expression. To clarify the mediators of the intergenerational regulation of Ocln, we performed small RNA-seq and focused on the role of miRNAs. Based on preliminary predictions using the miRwalk database and miRanda software on miRNA sequencing results, we identified miR-139-5p, miR-10b-5p, miR-200b-3p, and miR-200c-3p in the F1 colon as potential candidates that bind to the Ocln 3′ UTR. After further examining the expression of these four miRNAs in paternal colon and sperm, we identified miR-10b-5p and miR-200b-3p as two candidates that are likely to transmit epigenetic information. Subsequently, we validated the negative regulatory effects of miR-10b-5p and miR-200b-3p on the Ocln protein using a dual-luciferase reporter assay *in vitro*. Consistently, one study reported that inhibition of miR-200b-3p expression reduced intestinal mucosal injury and decreased mucosal permeability (55). Another study found that miR-200b expression was significantly upregulated in the serum of Crohn’s disease patients (56). Previously, multiple studies have focused on the role of miR-200b in preserving intestinal integrity to either inhibit epithelial-to-mesenchymal transition or promote IEC proliferation (57, 58). However, we acknowledge that our study has limitation in assessing the causal relationship between sperm-derived miR-10b-5p/miR-200b-3p and Ocln expression *in vivo*. In fact, direct microinjection of total small RNA from LONG paternal sperm, or synthetic miR-10b-5p and miR-200b-3p fragments, into wild-type zygotes is a classic method to study the transgenerational inheritance of sperm small RNA (59, 60). This will thoroughly assess the efficiency of sperm-derived miRNAs in regulating Ocln expression and inducing the protective intestinal phenotype in offspring. Overall, we observed the downregulation of miR-10b-5p and miR-200b-3p in the colon following antibiotic administration and demonstrated their direct regulatory relationship with Ocln expression.

A significant negative correlation was identified between paternal gut-enriched probiotics (*L. gasseri* and *P. merdae*) and paternal colonic miRNAs, suggests that the microbiota is involved in regulating miRNA expression. Based on the results that *L. gasseri* and *P. merdae* did not exhibit consistent alterations in the paternal and offspring gut, indicating that the microbiota did not directly act on the offspring’s gut. Substantial evidence indicates that microbiota-derived metabolites and derivatives, including indole-3-propionic acid, indole-3-lactic acid, and urolithins, modulate diverse miRNA levels, participating in the pathogenesis or therapeutic intervention (61–63). This suggests that the changes in microbial abundance observed in our study might similarly modulate epigenetic marks and miRNA expression via altered metabolite profiles, ultimately influencing gut barrier integrity. However, which metabolite mediates the regulatory processes in this study need to further study.

Notably, the protective phenotype was more pronounced in male offspring, potentially suggesting the involvement of sex hormone levels in regulating the intestinal response to epigenetic signals. Previous studies reported that estrogen receptor β has been shown to enhance intestinal epithelial barrier function, including upregulating the expression of tight junction proteins such as Occludin (64). Moreover, estrogen increases miR-10b-5p expression in T cells, and upregulates of miR-200b expression in MCF-7 breast cancer cells (65, 66). These suggest sex hormones are critical media between miRNA biology and intestinal homeostasis. Although this study did not directly examine the regulation of miR-10b-5p and miR-200b-3p by sex hormones, we speculate that estrogen may alter the inherited levels of these miRNAs in F1 female mice, thereby enhancing the gene silencing effect on Ocln.

Additionally, the resistant strains could have multifaceted impacts on gut homeostasis. For example, overgrowth of *Enterococcus* is closely associated with intestinal inflammation, and certain species can exacerbate colitis by impairing intestinal epithelial regeneration and inducing pro-inflammatory cytokine production (67, 68). Therefore, whether these drug-resistant bacterial strains would be vertically transmitted to the offspring, and associated effects on the intestinal barrier function and inflammatory response of the offspring, need to further study.

In summary, accumulating evidence indicates that ampicillin and cefixime administration starting from the weaning period reduces the levels of miR-10b-5p and miR-200b-3p in the colons of adult fathers, thereby increasing Ocln expression and improving colitis. This protective colitis phenotype is transmitted to male offspring through epigenetic inheritance of miRNAs in the sperm. Although our study describes a possible mechanism for the intergenerational transmission of the protective colitis phenotype between paternal and offspring generations, the mechanisms underlying decreased miR-10b-5p and miR-200b-3p expression following early antibiotic administration remain to be elucidated. Overall, these findings provide new insights into the intergenerational effects of early antibiotic administration, and highlight the importance of the weaning period for long-term gut health and disease risk.

## Data Availability

The datasets presented in this study can be found in online repositories. The names of the repository/repositories and accession number(s) can be found below: PRJNA1303027 and PRJNA1303393 (SRA).
