# Supplementary material for: Antibiotics administration during weaning ameliorates intestinal mucosal inflammation in adult mice and their offspring
Source: Front Immunol. 2026 Feb 26;17:1741596. doi: 10.3389/fimmu.2026.1741596 (PMC12979476; doi:10.3389/fimmu.2026.1741596)
Supplement: Supplementary file 1 [file DataSheet1.pdf]

Supplementary materials

Supplementary figures and figure legends

Supplementary Figure 1. Effect of antibiotic administration on intestinal epithelial cell function in weaned male mice under physiological condition

(A) Relative mRNA levels of *Alpi*, *Muc2*, *Defa5* and *ChgA* in distal ileum were evaluated by qRT-PCR. n=5-7 mice per group. (B-E) Immunofluorescence staining of Alpi, Muc2, Defa5 and ChgA in distal ileum. Quantitative analysis of the results was performed using Image J. n = 3-5 mice per group. Scale bar, 50 μm (B-D) and 100 μm (E). (F) Relative mRNA level of *Muc2* in colon was evaluated by qRT-PCR. n=5-7 mice per group. (G) Immunofluorescence staining of Muc2 in colon. Quantitative analysis of the results was performed using Image J. n=4 mice per group. Scale bar, 50 μm. Data are expressed as the mean ± SEM. Statistical significance was determined using one-way ANOVA followed by Tukey's multiple comparisons test; **P* < 0.05 and ****P* < 0.001.

Supplementary Figure 2. Birth indices and body weight in offspring

(A) Schematic diagram of F1 offspring generated by co-housing antibiotic-treated or control male mice with untreated 8-week-old females, respectively. (B) Number of pups per litter. CON, n = 13 litters; SHORT, n = 11 litters, LONG, n = 12 litters. (C) Pregnancy rate of females after by mating with antibiotic-treated or control male mice. (D) Body weight of F1 offspring were

measured every week since 3-week-old. CON, n = 87; SHORT, n =70; LONG, n =79. Data are expressed as the mean ± SEM.

Supplementary Figure 3. Effect of intestinal barrier markers in F1 male mice

(A and B) Colon length and H&E staining. n=4-7 mice per group. Scale bar, 100 μm (down) and 250 μm (upper). (C) Relative mRNA levels of *Tjp1*, *Ocln*, and *Cldn3* in colon were evaluated by qRT-PCR. n=4-7 mice per group. (D) Expression levels of ZO-1, Ocln and Cldn in colon were evaluated by western blot. Quantitative analysis of the results was performed using Image J. n=4 mice per group. Data are expressed as the mean ± SEM. Statistical significance was determined using one-way ANOVA followed by Tukey's multiple comparisons test; *$P < 0.05$, **$P < 0.01$ and ***$P < 0.001$.

Supplementary Figure 4. Effect of intestinal epithelial cell function in F1 male mice

(A) Relative mRNA levels of *Alpi*, *Muc2*, *Defa5* and *ChgA* in distal ileum of F1 male mice were evaluated by qRT-PCR. n=4-7 mice per group. (B-E) Immunofluorescence staining of Alpi, Muc2, Defa5 and ChgA in distal ileum. Quantitative analysis of the results was performed using Image J. n = 3-5 mice per group. Scale bar, 50 μm (B-D) and 100 μm (E). (F) Relative mRNA level of *Muc2* in colon was evaluated by qRT-PCR. n=4-7 mice per group. (G) Immunofluorescence staining of Muc2 in colon. Quantitative analysis of the results was performed using Image J. n=4-5 mice per group. Scale bar, 50 μm.

Data are expressed as the mean ± SEM. Statistical significance was determined using one-way ANOVA followed by Tukey's multiple comparisons test; *P < 0.05 and **P < 0.01.

Supplementary Figure 5. Effect of of intestinal barrier markers in F1 female mice

(A and B) Colon length and H&E staining. n=7 mice per group. Scale bar, 100 μm (down) and 250 μm (upper). (C) Relative mRNA levels of *Tjp1*, *Ocln*, and *Cldn3* in colon were evaluated by qRT-PCR. n=5 mice per group. (D) Expression levels of ZO-1, Ocln, and Cldn in colon were evaluated by western blot. Quantitative analysis of the results was performed using Image J. n=4 mice per group. Data are expressed as the mean ± SEM. Statistical significance was determined using one-way ANOVA followed by Tukey's multiple comparisons test; *P < 0.05.

Supplementary Figure 6. Effect of intestinal epithelial cell function in F1 female mice

(A) Relative mRNA levels of *Alpi*, *Muc2*, *Defa5* and *ChgA* in distal ileum of F1 female mice were evaluated by qRT-PCR. n=5 mice per group. (B-E) Immunofluorescence staining of Alpi, Muc2, Defa5 and ChgA in distal ileum. Quantitative analysis of the results was performed using Image J. n=5 mice per group. Scale bar, 50 μm (B-D) and 100 μm (E). (F) Relative mRNA level of *Muc2* in colon was evaluated by qRT-PCR. n=5 mice per group. (G) Immunofluorescence staining of Muc2 in colon. Quantitative analysis of the

results was performed using Image J. n=5 mice per group. Scale bar, 50 μm. Data are expressed as the mean ± SEM. Statistical significance was determined using one-way ANOVA followed by Tukey's multiple comparisons test; *$P < 0.05$.

Supplementary Figure 7. Effect of DSS-induced colitis in F1 female offspring

(A and B) Body weight and diarrhea scores of F1 female mice were examined every day during the course of DSS treatment. n=6-7 mice per group. (C) Gross morphology and length of the colon in different groups. n=6-7 mice per group. (D) Colonic H&E staining at Day 9 after DSS challenge. The right panel is the histological scores. n=3 mice per group. Scale bar, 100 μm (down) and 250 μm (upper). (E) Relative mRNA levels of *Il1b, Il6, Il17a*, and *Tnfa* in distal colon were evaluated by qRT-PCR. n=6-7 mice per group. (F) Relative mRNA levels of *Tjp1*, *Ocln*, and *Cldn3* in distal colon were evaluated by qRT-PCR. n=6-7 for each group. Data are expressed as the mean ± SEM. Statistical significance was determined using one-way or two-way ANOVA followed by Tukey's multiple comparisons test; *$P < 0.05$, **$P < 0.01$ and ***$P < 0.001$.

Supplementary Figure 8. KEGG analysis of targets of differential expressed miRNAs

(A and B) The target genes of differential expressed miRNAs were predicted using miRWalk and miRTarBase databases. A total of 977 target genes were obtained, including 406 upregulated miRNA target genes and 571

downregulated miRNA target genes. KEGG enrichment of the upregulated and downregulated miRNA target genes, respectively.

Supplementary Figure 9. Correlation analysis between *Lactobacillus gasseri* / *Parabacteroides merdae* and miRNAs expression in paternal and offspring

(A) Spearman correlation analysis between probiotic abundance (*L. gasseri* and *P. merdae*) and miRNAs expression (miR-10b-5p and miR-200b-3p) in the paternal intestine under physiological conditions, respectively. n = 10 mice. (B and C) Relative abundance of *L. gasseri* and *P. merdae* in the cecum of F1 male and female mice was evaluated by qRT-PCR. n=7 mice per group. (D) Spearman correlation analysis between probiotic abundance and miRNAs expression in the F1 male mice intestine under physiological conditions, respectively. n = 14 mice. Data are expressed as the mean ± SEM. Statistical significance was determined using Wilcoxon rank-sum test; *P < 0.05, **P < 0.01 and ***P < 0.001.

Supplementary Figure 10. Resistant-strain evaluation

Stool samples were homogenized in sterile anaerobic PBS, was filtered and 92 diluted 10^2-fold. Then, 100 ul dilution was added to BHI agar plates containing ampicillin and cefixime, and cultured aerobically or anaerobically at 37 ^{o}C for 48 h. (A) Images of resistant-strains of CON and LONG mice. (B) the resistant-strains and their proportion under aerobic or anaerobic culture, respectively.

Supplementary tables

Supplementary Table 1. RT-qPCR primers

Genes	Species	Primer	Sequence (5′-3′)
Il1b	Mouse	Forward	GAAATGCCACCTTTTGACAGTG
		Reverse	TGGATGCTCTCATCAGGACAG
Il6	Mouse	Forward	TAGTCCTTCCTACCCCAATTTCC
		Reverse	TTGGTCCTTAGCCACTCCTTC
Il17a	Mouse	Forward	TTTAACTCCCTTGGCGCAAAA
		Reverse	CTTTCCCTCCGCATTGACAC
Tnfa	Mouse	Forward	AAGCCTGTAGCCCACGTCGTA
		Reverse	AGGTACAACCCATCGGCTGG
Tjp1	Mouse	Forward	GCCGCTAAGAGCACAGCAA
		Reverse	TCCCCACTCTGAAAATGAGGA
Ocln	Mouse	Forward	TGAAAGTCCACCTCCTTACAGA
		Reverse	CCGGATAAAAAGAGTACGCTGG
Cldn3	Mouse	Forward	ACCAACTGCGTACAAGACGAG
		Reverse	CGGGCACCAACGGGTTATAG
Alpi	Mouse	Forward	AGGACATCGCCACTCAACTC
		Reverse	GGTTCCAGACTGGTTACTGTCA
Muc2	Mouse	Forward	ATGCCCACCTCCTCAAAGAC
		Reverse	GTAGTTTCCGTTGGAACAGTGAA
Defa5	Mouse	Forward	AGGCTGATCCTATCCACAAAACAG
		Reverse	TGAAGAGCAGACCCTTCTTGGC
Chga	Mouse	Forward	TCTGCCGTCTGAAGGGAAG
		Reverse	TCCTGCTTATGTTCCAGCTCC
Gapdh	Mouse	Forward	TGTGTCCGTCGTGGATCTGA
		Reverse	CCTGCTTCACCACCTTCTTGAT
OCLN	Human	Forward	GACTTCAGGCAGCCTCGTTAC
		Reverse	GCCAGTTGTGTAGTCTGTCTCA
GAPDH	Human	Forward	CTGGGCTACACTGAGCACC
		Reverse	AAGTGGTCGTTGAGGGCAATG
miR-139-5p	Mouse	Forward	CTGGTAGGTCTACAGTGCAC
		Reverse	CTCAACTGGTGTCGTGGAGT
miR-10b-5p	Mouse	Forward	CTGGTAGGTACCCTGTAGAA
		Reverse	CTCAACTGGTGTCGTGGAGT
miR-200b-3p	Mouse	Forward	CTGGTAGGTAATACTGCCTG
		Reverse	CTCAACTGGTGTCGTGGAGT
miR-200c-3p	Mouse	Forward	CTGGTAGGTAATACTGCCG
		Reverse	CTCAACTGGTGTCGTGGAGT
U6	Mouse	Forward	CTCGCTTCGGCAGCACA
		Reverse	AACGCTTCACGAATTTGCGT

Supplementary Table 2. miRNA reverse transcription primers

Genes	Primer	Sequence (5′-3′)
miR-139-5p	Reverse transcription primer	CTCAACTGGTGTCGTGGAGTCG GCAATTCAGTTGAGCTGGAGAC
miR-10b-5p	Reverse transcription primer	CTCAACTGGTGTCGTGGAGTCG GCAATTCAGTTGAGCACAAATT
miR-200b-3p	Reverse transcription primer	CTCAACTGGTGTCGTGGAGTCG GCAATTCAGTTGAGTCATCATT
miR-200c-3p	Reverse transcription primer	CTCAACTGGTGTCGTGGAGTCG GCAATTCAGTTGAGTCCATCAT

Supplementary Table 3. Sequences of miRNA mimics

Primer	Sequence (5’-3’)
Negative control sense	UUCUCCGAACGUGUCACGUTT
Negative control antisense	ACGUGACACGUUCGGAGAATT
miR-10b-5p mimics sense	UACCCUGUAGAACCGAAUUUGUG
miR-10b-5p mimics antisense	CAAAUUCGGUUCUACAGGGUAUU
miR-200b-3p mimics sense	UAAUACUGCCUGGUAAUGAUGA
miR-200b-3p mimics antisense	AUCAUUACCAGGCAGUAUUAUU

Supplementary Table 4. Primers for vector construction

Primer	Sequence (5’-3’)
mOcln-3’UTR-F	CCCTCGAGGGAGAGATGCCAGTTGCGGGAGA
mOcln-3’UTR-R	TTGCGGCCGCAACACACACACGTGCGCATC
Ocln-10b-mut-F	TTCCAGCCTGAGCTTCTCTGTGTAGCCCTGGCTG
Ocln-10b-mut-R	AGAAGCTCAGGCTGGAAAACCAAAAACAACCTGA
Ocln-200b-mut-F	ATCTCGATTCTAGAAGCATTTTATAAATGGCTTTTGAT
Ocln-200b-mut-R	GCTTCTAGAATCGAGATAGGAAACTGATGGAATTAAA GC

Fig.S1

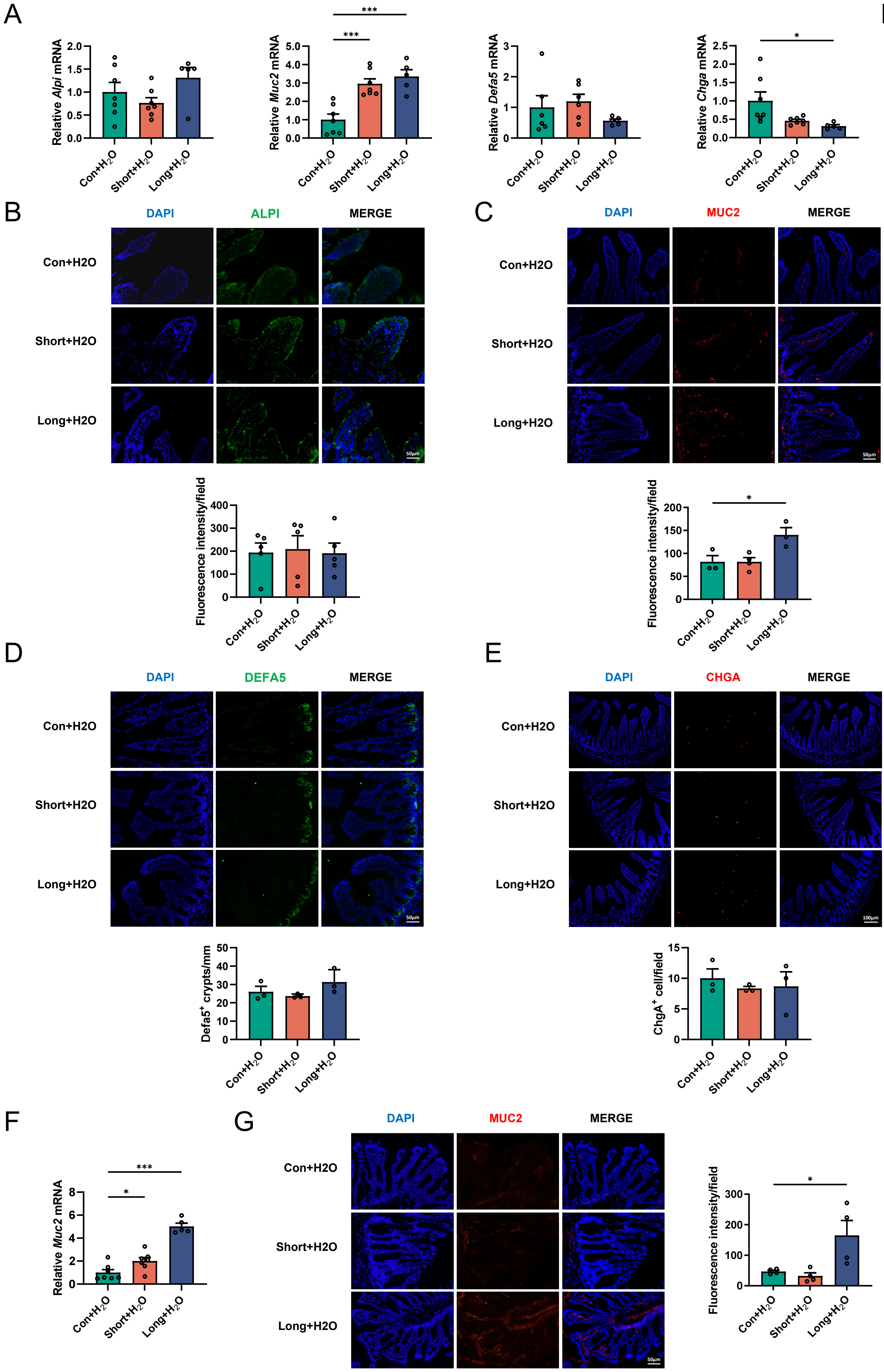
A
Relative Alpi mRNA
Relative Muc2 mRNA
Relative Defa5 mRNA
Relative Chga mRNA
***
*
Con+H_2O
Short+H_2O
Long+H_2O
B
DAPI
ALPI
MERGE
Con+H2O
Short+H2O
Long+H2O
50μm
Fluorescence intensity/field
C
DAPI
MUC2
MERGE
D
DAPI
DEFA5
MERGE
Defa5+ crypts/mm
E
DAPI
CHGA
MERGE
100μm
ChgA+ cell/field
F
Relative Muc2 mRNA
G
DAPI
MUC2
MERGE
Fluorescence intensity/field

Fig.S2

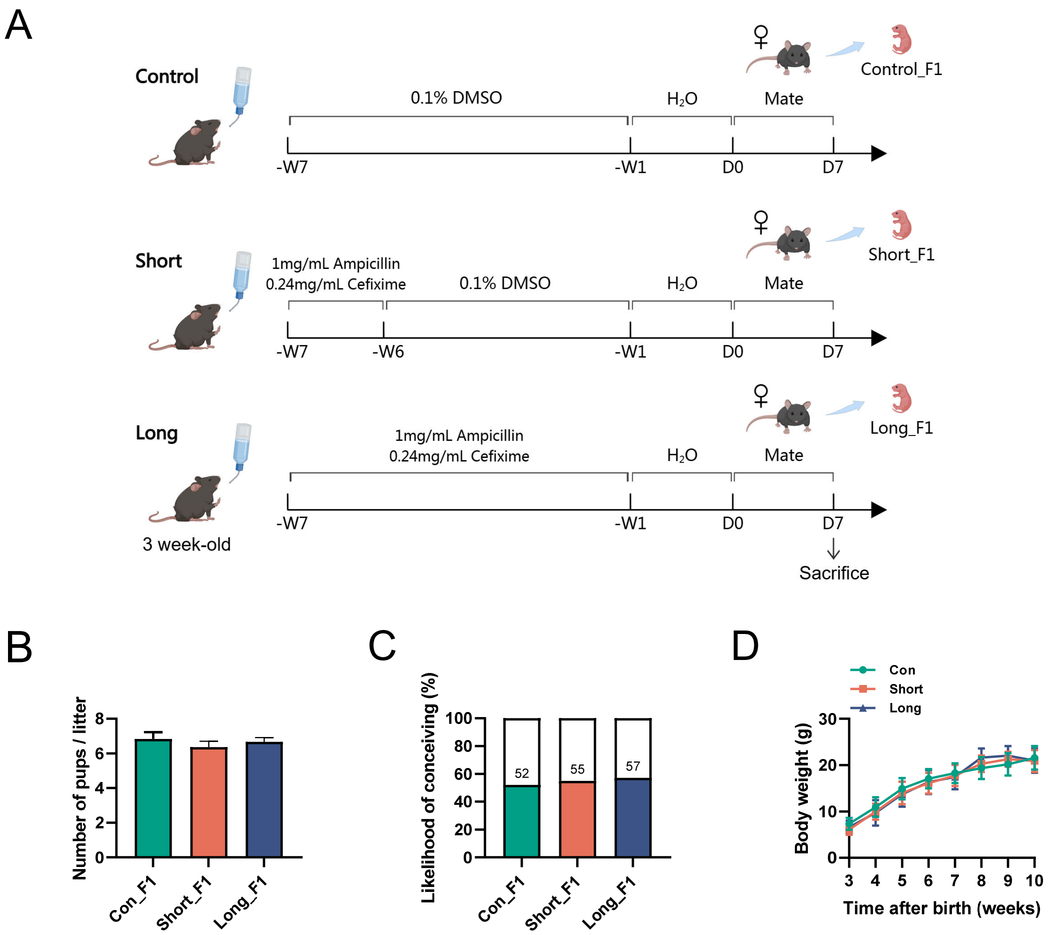

A
Control
0.1% DMSO
H_2O
Mate
Control_F1
-W7
-W1
D0
D7
Short
1mg/mL Ampicillin
0.24mg/mL Cefixime
0.1% DMSO
H_2O
Mate
Short_F1
-W7
-W6
-W1
D0
D7
Long
1mg/mL Ampicillin
0.24mg/mL Cefixime
H_2O
Mate
Long_F1
3 week-old
-W7
-W1
D0
D7
Sacrifice
B
Number of pups / litter
Con_F1
Short_F1
Long_F1
C
Likelihood of conceiving (%)
52
55
57
Con_F1
Short_F1
Long_F1
D
Con
Short
Long
Body weight (g)
Time after birth (weeks)

Fig.S3

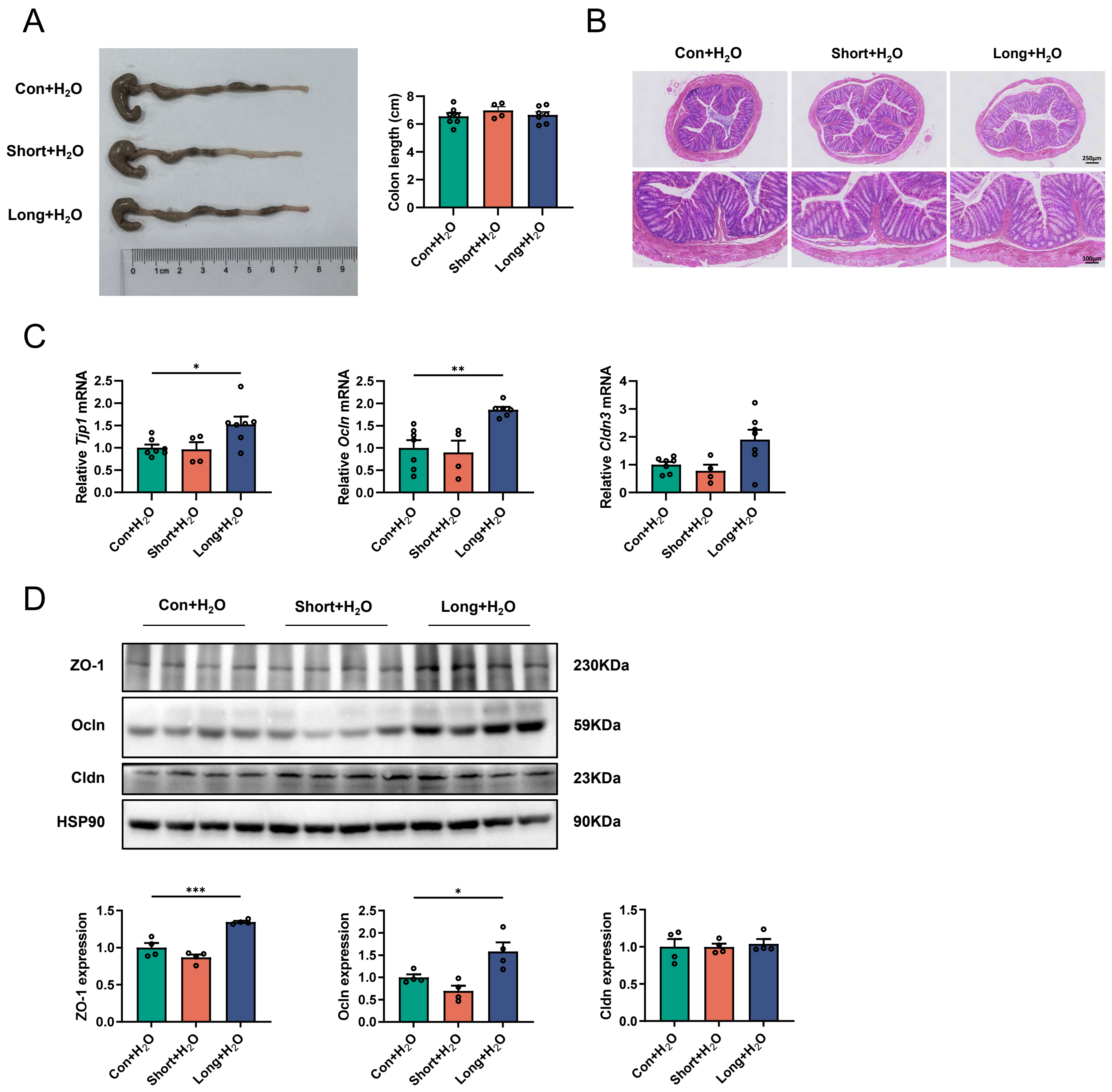

A
Con+H2O
Short+H2O
Long+H2O
Colon length (cm)
B
Con+H2O
Short+H2O
Long+H2O
C
Relative Tjp1 mRNA
Relative Ocln mRNA
Relative Cldn3 mRNA
D
Con+H2O
Short+H2O
Long+H2O
ZO-1
Ocln
Cldn
HSP90
230KDa
59KDa
23KDa
90KDa
ZO-1 expression
Ocln expression
Cldn expression

Fig.S4

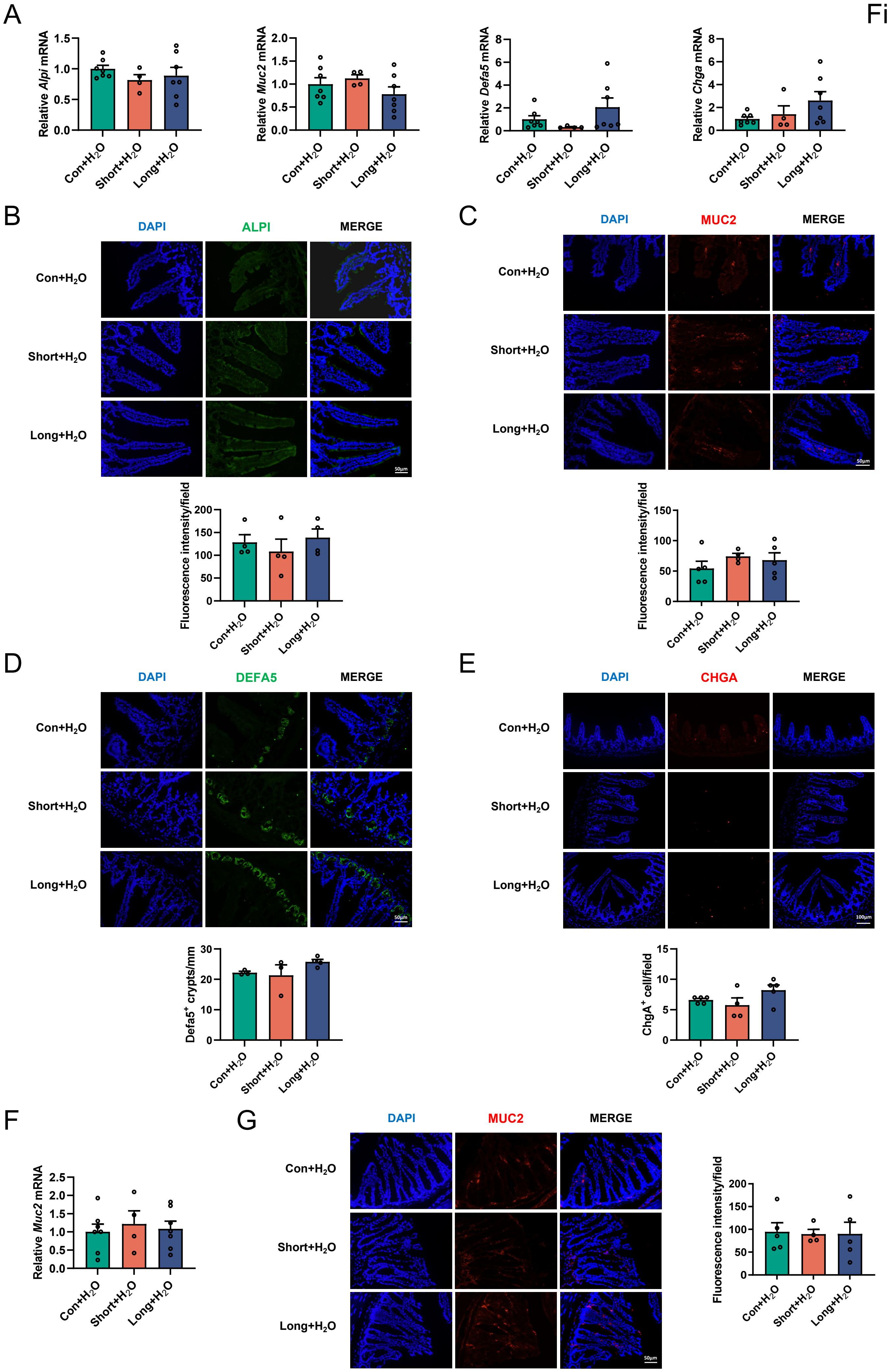
A
Relative Alpi mRNA
Relative Muc2 mRNA
Relative Defa5 mRNA
Relative Chga mRNA
Con+H2O
Short+H2O
Long+H2O
B
DAPI
ALPI
MERGE
50μm
Fluorescence intensity/field
C
MUC2
D
DEFA5
Defa5+ crypts/mm
E
CHGA
100μm
ChgA+ cell/field
F
G

Fig.S5

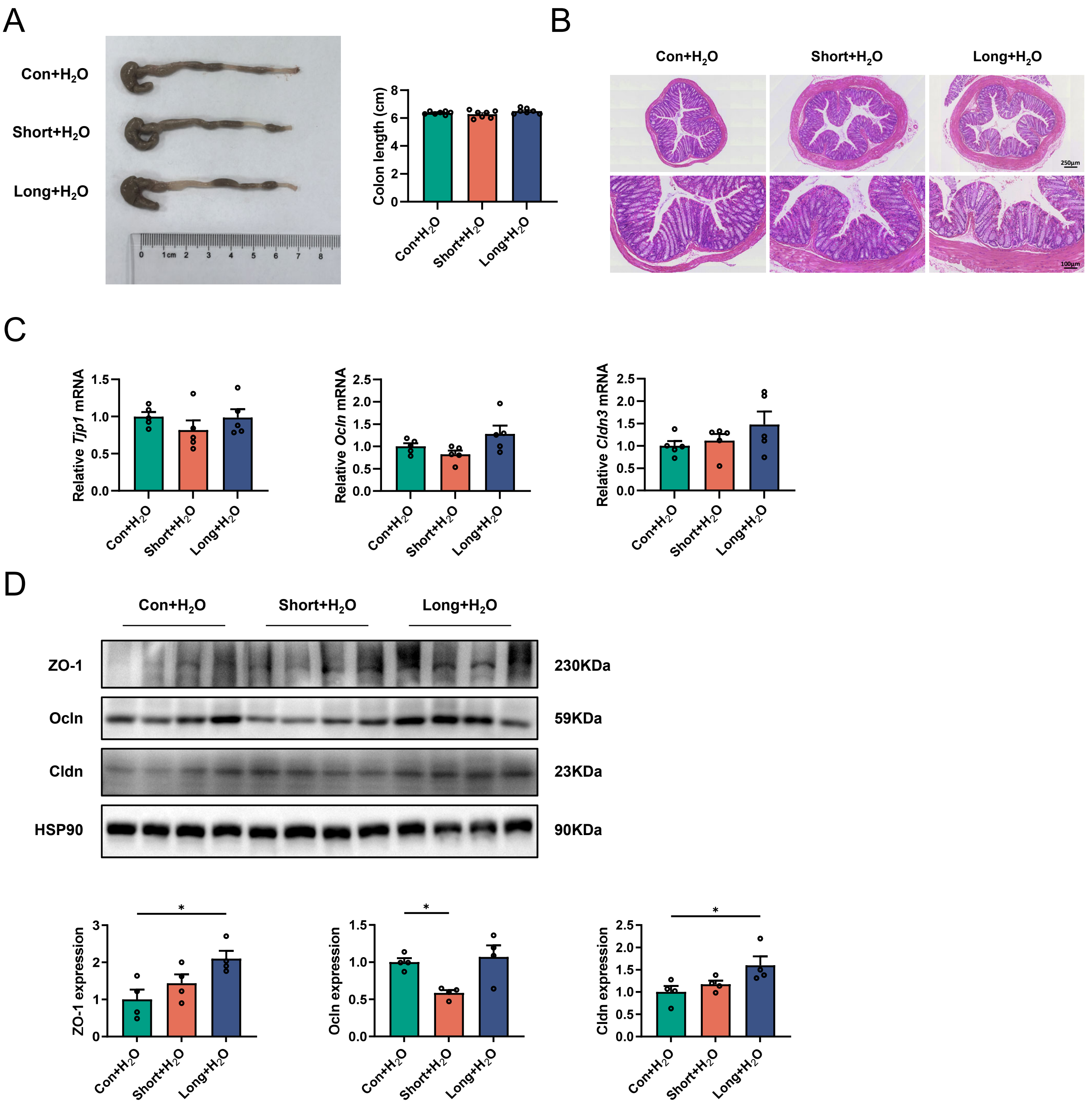
A
Con+H2O
Short+H2O
Long+H2O
Colon length (cm)
B
C
Relative Tjp1 mRNA
Relative Ocln mRNA
Relative Cldn3 mRNA
D
ZO-1
Ocln
Cldn
HSP90
230KDa
59KDa
23KDa
90KDa
ZO-1 expression
Ocln expression
Cldn expression
250μm
100μm

Fig.S6

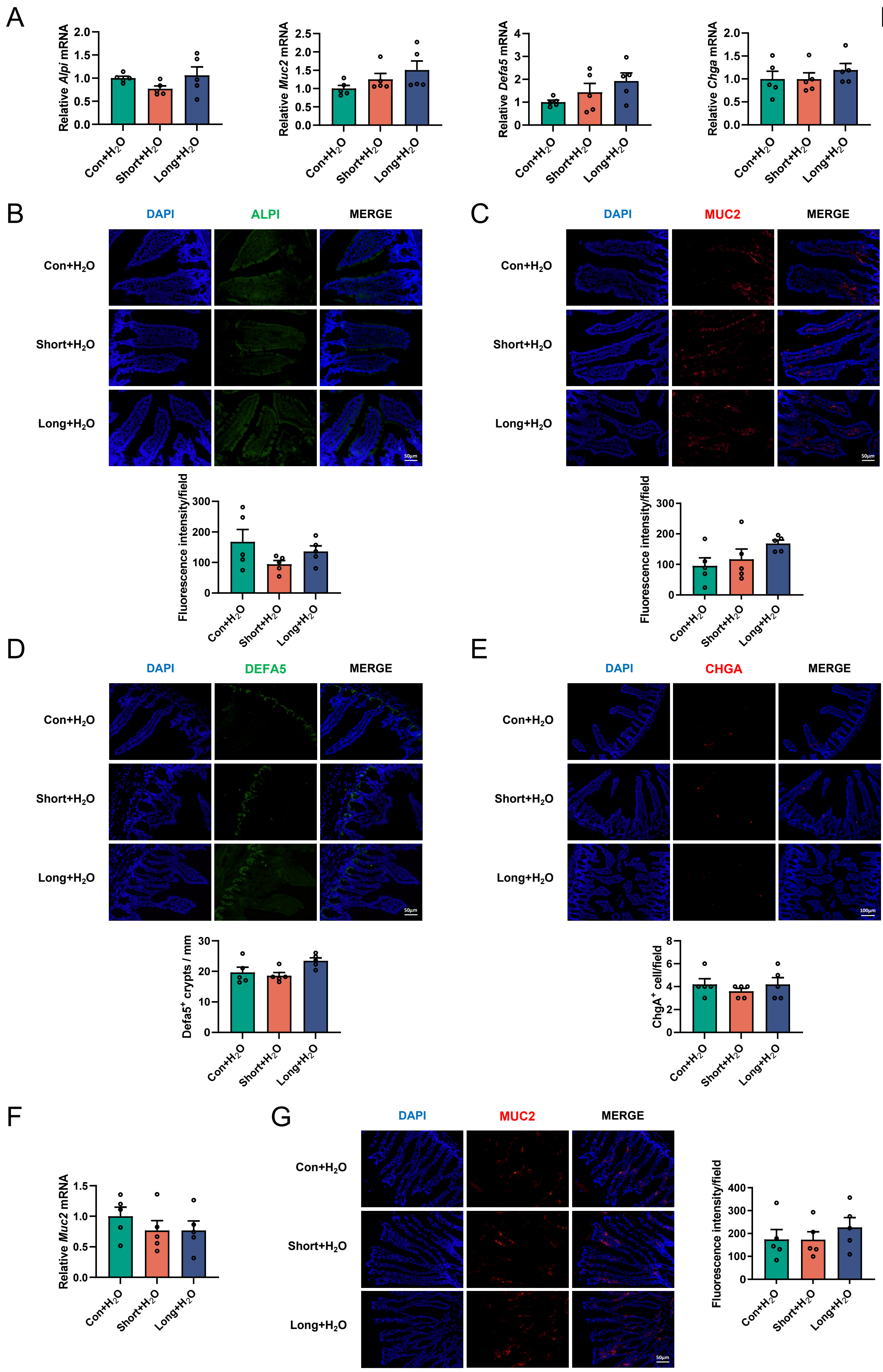
A
Relative Alpi mRNA
Relative Muc2 mRNA
Relative Defa5 mRNA
Relative Chga mRNA
Con+H2O
Short+H2O
Long+H2O
B
DAPI
ALPI
MERGE
C
MUC2
D
DEFA5
E
CHGA
F
G
Fluorescence intensity/field
Defa5+ crypts / mm
ChgA+ cell/field
50μm
100μm

Fig.S7

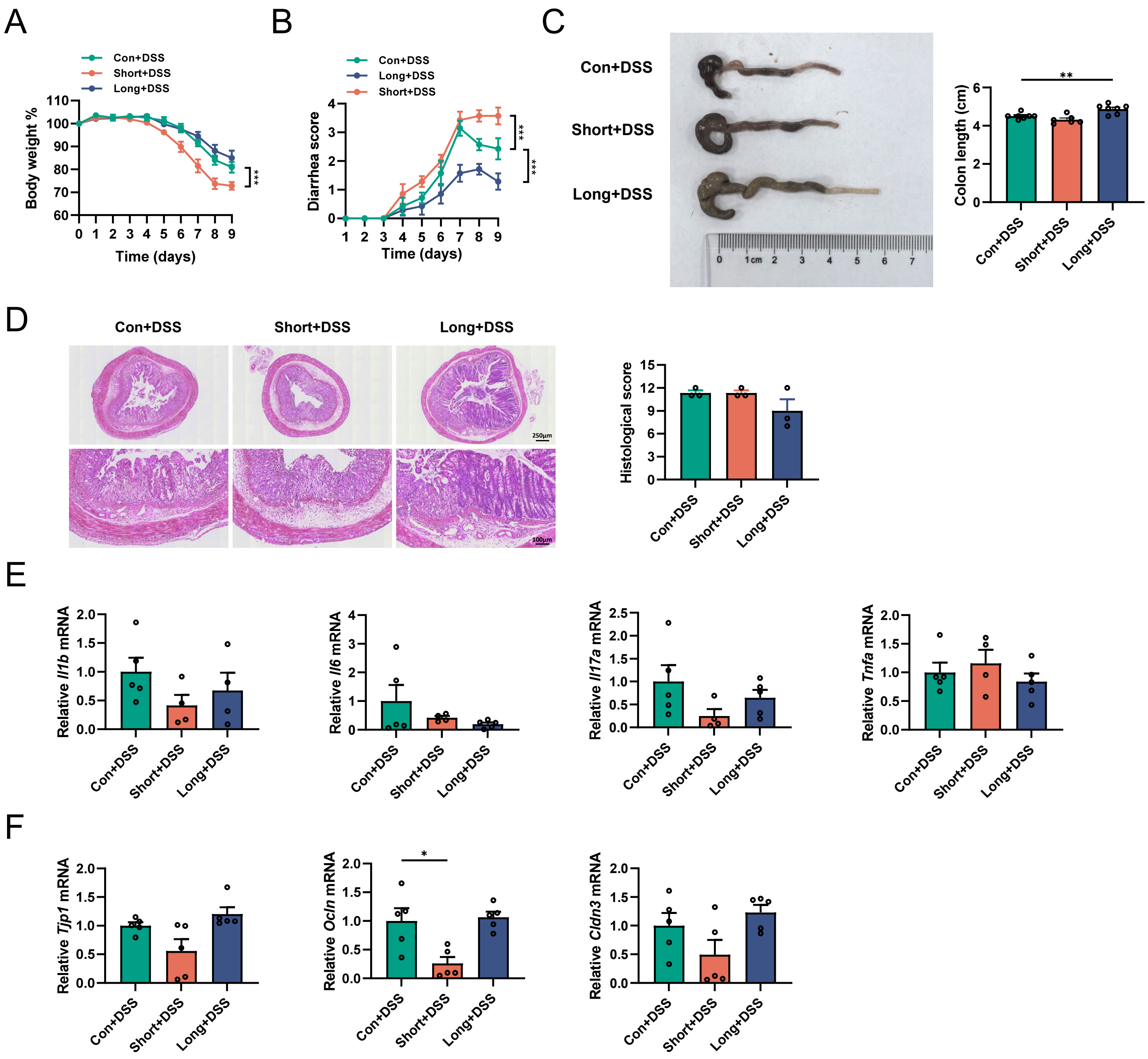

A
Con+DSS
Short+DSS
Long+DSS
Body weight %
Time (days)
***
B
Con+DSS
Long+DSS
Short+DSS
Diarrhea score
Time (days)
***
***
C
Con+DSS
Short+DSS
Long+DSS
Colon length (cm)
**
D
Con+DSS
Short+DSS
Long+DSS
250μm
100μm
Histological score
E
Relative Il1b mRNA
Relative Il6 mRNA
Relative Il17a mRNA
Relative Tnfa mRNA
F
Relative Tjp1 mRNA
Relative Ocln mRNA
*
Relative Cldn3 mRNA

Fig.S8

A

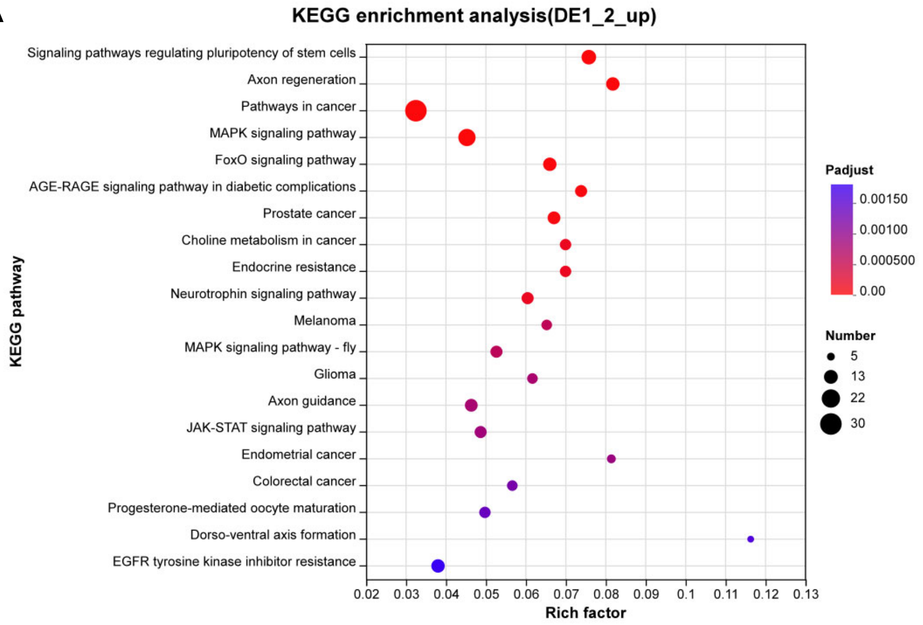
KEGG enrichment analysis(DE1_2_up)
Signaling pathways regulating pluripotency of stem cells
Axon regeneration
Pathways in cancer
MAPK signaling pathway
FoxO signaling pathway
AGE-RAGE signaling pathway in diabetic complications
Prostate cancer
Choline metabolism in cancer
Endocrine resistance
Neurotrophin signaling pathway
Melanoma
MAPK signaling pathway - fly
Glioma
Axon guidance
JAK-STAT signaling pathway
Endometrial cancer
Colorectal cancer
Progesterone-mediated oocyte maturation
Dorso-ventral axis formation
EGFR tyrosine kinase inhibitor resistance
KEGG pathway
0.02 0.03 0.04 0.05 0.06 0.07 0.08 0.09 0.1 0.11 0.12 0.13
Rich factor
Padjust
0.00150
0.00100
0.000500
0.00
Number
5
13
22
30

B

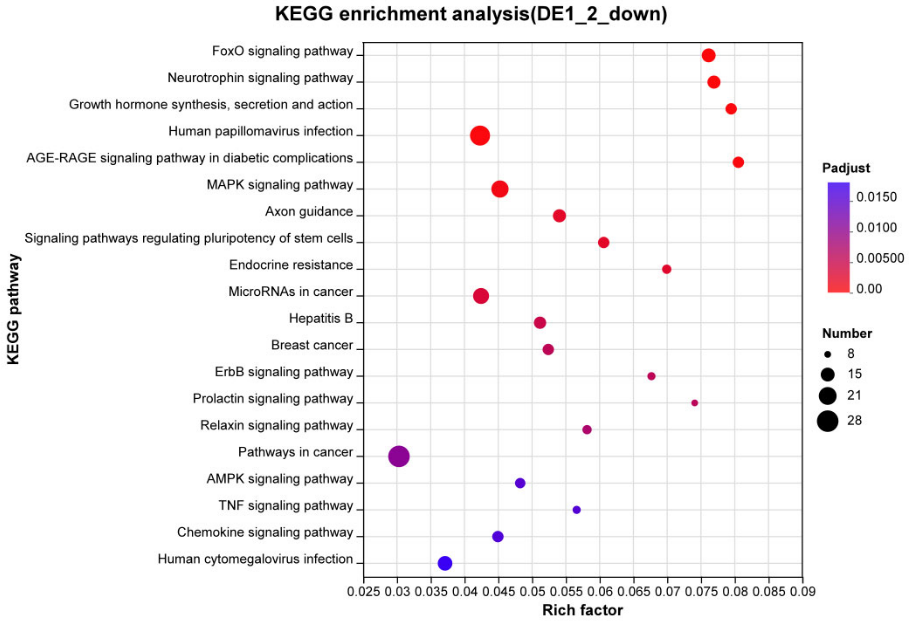
KEGG enrichment analysis(DE1_2_down)
FoxO signaling pathway
Neurotrophin signaling pathway
Growth hormone synthesis, secretion and action
Human papillomavirus infection
AGE-RAGE signaling pathway in diabetic complications
MAPK signaling pathway
Axon guidance
Signaling pathways regulating pluripotency of stem cells
Endocrine resistance
MicroRNAs in cancer
Hepatitis B
Breast cancer
ErbB signaling pathway
Prolactin signaling pathway
Relaxin signaling pathway
Pathways in cancer
AMPK signaling pathway
TNF signaling pathway
Chemokine signaling pathway
Human cytomegalovirus infection
KEGG pathway
0.025 0.03 0.035 0.04 0.045 0.05 0.055 0.06 0.065 0.07 0.075 0.08 0.085 0.09
Rich factor
Padjust
0.0150
0.0100
0.00500
0.00
Number
8
15
21
28

Fig.S9

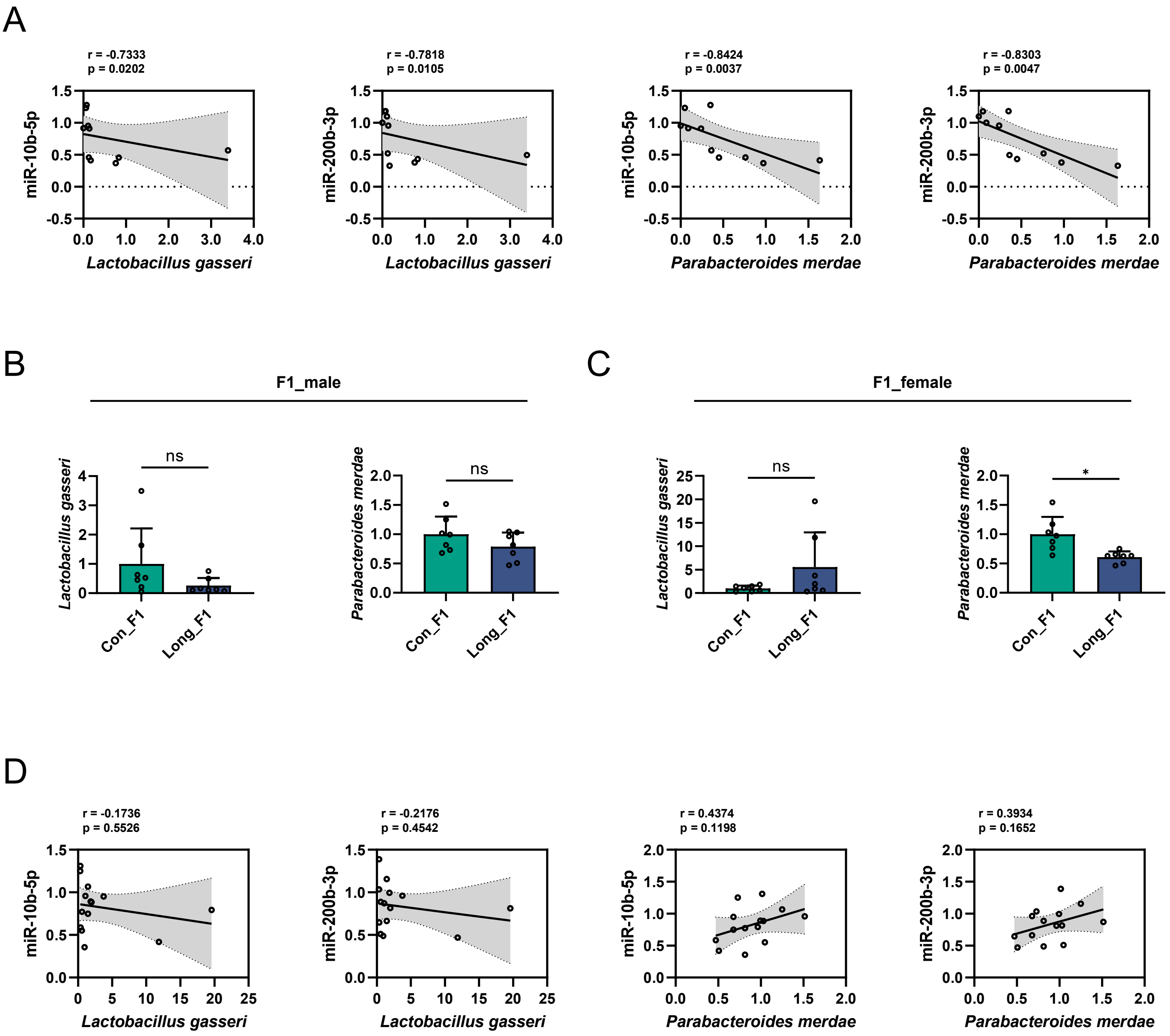
A
r = -0.7333
p = 0.0202
miR-10b-5p
Lactobacillus gasseri
r = -0.7818
p = 0.0105
miR-200b-3p
Lactobacillus gasseri
r = -0.8424
p = 0.0037
miR-10b-5p
Parabacteroides merdae
r = -0.8303
p = 0.0047
miR-200b-3p
Parabacteroides merdae
B
F1_male
Lactobacillus gasseri
ns
Parabacteroides merdae
ns
Con_F1
Long_F1
C
F1_female
Lactobacillus gasseri
ns
Parabacteroides merdae
*
Con_F1
Long_F1
D
r = -0.1736
p = 0.5526
miR-10b-5p
Lactobacillus gasseri
r = -0.2176
p = 0.4542
miR-200b-3p
Lactobacillus gasseri
r = 0.4374
p = 0.1198
miR-10b-5p
Parabacteroides merdae
r = 0.3934
p = 0.1652
miR-200b-3p
Parabacteroides merdae

Fig.S10

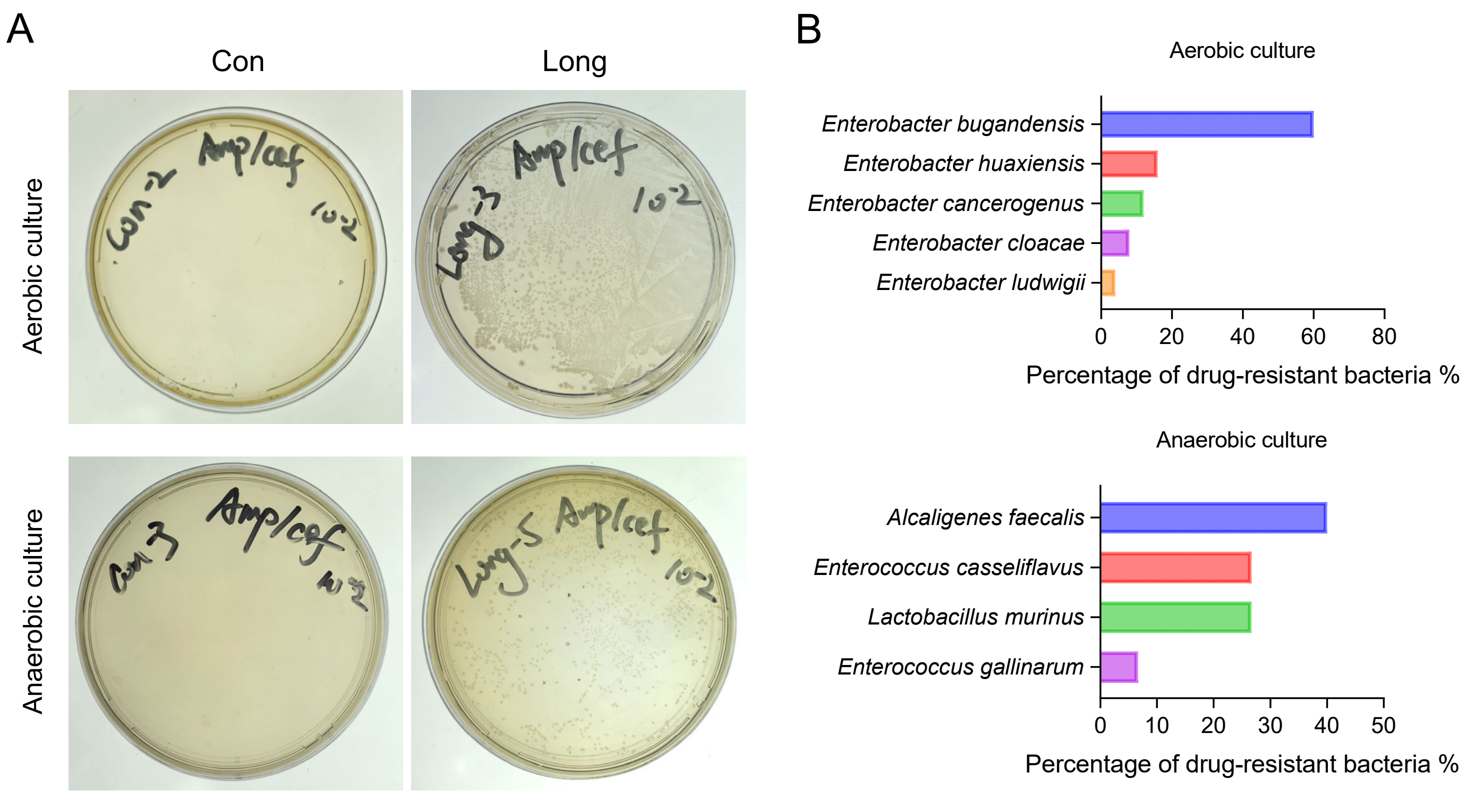
A
Con
Long
Aerobic culture
Anaerobic culture
B
Aerobic culture
Enterobacter bugandensis
Enterobacter huaxiensis
Enterobacter cancerogenus
Enterobacter cloacae
Enterobacter ludwigii
0 20 40 60 80
Percentage of drug-resistant bacteria %
Anaerobic culture
Alcaligenes faecalis
Enterococcus casseliflavus
Lactobacillus murinus
Enterococcus gallinarum
0 10 20 30 40 50
Percentage of drug-resistant bacteria %
